# Associations between indoor environmental quality in schools and symptom reporting in pupil-administered questionnaires

**DOI:** 10.1186/s12940-019-0555-6

**Published:** 2019-12-27

**Authors:** Kateryna Savelieva, Tero Marttila, Jussi Lampi, Sari Ung-Lanki, Marko Elovainio, Juha Pekkanen

**Affiliations:** 1 Department of Public Health, Faculty of Medicine, University of Helsinki, 00014 Helsinki, Finland; 2 Unit of Civil Engineering, Faculty of Built Environment, Tampere University, 33014 Tampere, Finland; 3 Department of Health Security, Environmental Health, National Institute for Health and Welfare, 70701 Kuopio, Finland; 4 Department of Psychology and Logopedics, Faculty of Medicine, University of Helsinki, 00014 Helsinki, Finland

**Keywords:** Indoor environmental quality, Symptom reporting, Respiratory symptoms, School, Questionnaire, Child health

## Abstract

**Background:**

The associations between indoor environmental quality (IEQ) in homes and symptom reporting of children have been extensively studied, but only few large-scale studies have been done in schools. We examined associations between expert-assessed IEQ in schools and pupils’ reporting of different symptoms, and whether associations were stronger if participants relate symptoms to the school environment.

**Methods:**

The questionnaire survey was done in all primary and secondary schools in two areas of Helsinki, Finland. Primary school pupils (grade 3–6, *n* = 8775, 99 school-buildings) and secondary school pupils (grade 7–9, *n* = 3410, 30 school-buildings) reported their symptoms. Symptoms were combined into respiratory, lower respiratory, eye, skin, and general symptom groups. Surveys were also done among the parents of the primary school pupils (grade 1–6, *n* = 3540, 88 school buildings), but results are reported only in the supplement due to the low response rate (20% in 2017 and 13% in 2018). The associations between IEQ and symptoms were analyzed using multilevel logistic regression analysis.

**Results:**

Several of the IEQ indicators were highly correlated and indicators were therefore mainly analyzed by combining them into a summary score and into latent classes. Dose-response associations were found between IEQ problems and higher reporting of respiratory and general symptoms among both primary and secondary school pupils. Some associations were also observed with lower respiratory and skin symptoms, but not with eye symptoms. The associations were somewhat stronger with symptoms related to the school environment compared to symptoms reported without such relation: for a unit change in IEQ summary score and respiratory symptoms in primary schools, odds ratios were 1.07 (95% CI 1.02–1.06) and 1.04 (95% CI 1.04–1.10), and in secondary schools 1.09 (95% CI 1.01–1.09) and 1.05 (95% CI 1.02–1.17), respectively.

**Conclusions:**

Expert-assessed IEQ problems in schools were associated with increased reporting of especially respiratory and general symptoms. The associations were only somewhat stronger in magnitude for symptoms reported in relation to the school environment compared to symptoms reported without such relation.

## Background

Indoor environmental quality (IEQ) problems are common in schools and may adversely influence the performance and attendance of pupils and increase symptom reporting [1–3]. IEQ is defined as the quality of building indoor environment and encompasses several environmental factors: indoor air quality (e.g., dampness and mold, odors), thermal conditions, acoustical quality, and lighting quality [3, 4]. The associations between various IEQ problems at home and adverse respiratory and allergic health effects in children have repeatedly been shown [5–11]; however, more research is needed to examine these associations in schools [12].

Previous studies have shown consistent associations between IEQ indicators, especially moisture and mold damage in schools, and a higher number of upper and lower respiratory symptoms of pupils [13–18]. Several studies have also found similar associations among teachers [19–21]. The evidence is sparse, however, regarding the associations between IEQ indicators and general symptoms (i.e., tiredness, headache, and difficulties concentrating), showing no such associations [18, 22] or small and nearly significant associations [23]. Some studies have also shown the associations between IEQ in schools and eye and skin symptoms among pupils [24–26].

Questionnaires on indoor air and related symptoms, which have been widely used in office places [27], are also used to survey the indoor environment and health of pupils in schools. Although not always, these questionnaires usually ask whether symptoms get worse in a certain indoor environment or even whether symptoms are attributed to (or blamed on) a certain environment [27]. Some respondents find it difficult to assess this, and symptoms that are attributed to a specific environment may also produce responses more related to environmental concerns [28], although this has not been studied, to our knowledge. There also appears to be no previous studies that would have examined whether the associations between IEQ and symptom reporting differ when symptoms are asked in relation to being in school and when symptoms are experienced in general (i.e., without relation to any specific environment).

In indoor environment-related questionnaires, parents’ reports are still used as a proxy for primary school pupils’ symptom reporting; although some studies have already started administrating questionnaires to primary school pupils [29]. There are, however, discrepancies between parents’ proxy reports and children’s self-reports on symptoms [30], and it has been recommended to use child-administered questionnaires about child’s symptoms and internal states [31, 32]. One recent study [32] has also reported that primary school pupils (i.e., aged 9–12 years) can provide reliable information about their symptoms and perception of indoor air, supporting the possibility to administer indoor environment-related questionnaires to pupils aged nine and above in schools in the future.

The current study draws on a large cross-sectional questionnaire survey data collected in all schools from two areas of Helsinki, Finland. The aim of the study was to examine the associations between school IEQ and reporting of different symptoms (respiratory, lower respiratory, eye, skin, and general) of primary and secondary school pupils, and whether these associations differ when symptoms are asked in relation to being in school and when symptoms are asked without relation to the school environment (experienced in general).

## Methods

### Study population

Cross-sectional data came from the survey of indoor environmental quality and symptom reporting, conducted in all primary (grade 1–6) and secondary schools (grade 7–9) in Helsinki, Finland. Helsinki was divided into three parts: the first survey was conducted in all schools in southern and central regions during spring 2016; the second survey in all schools in northern, western, and northeastern regions during winter 2017; and the third survey in all schools in eastern regions, as well as in all Swedish-speaking schools of Helsinki during winter 2018. The present study comprised data from the second and third surveys, because there was no expert evaluation of indoor environmental quality on the school-building level in the first survey. The majority of school buildings were built between 1950s and 1990s. Most school buildings have balanced mechanical ventilation, some have exhaust-only mechanical ventilation, and a few have natural (non-mechanical) ventilation.

The survey was conducted in 33 primary schools and 13 secondary schools in 2017 [33] and 43 primary schools and 23 secondary schools in 2018 [34]. In primary schools, all 3–6-grade pupils and parents of 1–6-grade pupils were invited to participate in the survey; in secondary schools, all 7–9-grade pupils were invited to participate. In primary and secondary schools, pupils filled in the questionnaires in classrooms via the electronic form under the teacher supervision. Participation in the survey was voluntary, and parents could refuse the use of their children’s information both for primary and secondary school pupils. Only six parents refused the use of their children’s information: out of these six instances, five pupils did not fill in the questionnaire administered in the school; one pupil has filled in the questionnaire, but it had been withdrawn from the study. The research plans were approved by the Institutional Review Board of National Institute for Health and Welfare (THL), Finland (THL/1370/6.02.01/2016).

The response rate of primary and secondary school pupils was in general above 50% in 2017 and 2018; whereas the response rate of parents was very low (20% in 2017 and 13% in 2018) (Additional file 1: Table S1). Because of the low parental response rate, we did not conduct any main analyses with parental data and used it only to provide supplementary evidence for our study aims.

For this study, we combined data from 2017 and 2018 surveys, which resulted in the following number of participants (and school buildings): 9835 3–6-grade pupils (121 school buildings) and 3965 7–9-grade pupils (38 school buildings). We excluded the schools with special education and the school buildings in which there were less than 10 responses per building or missing expert evaluation of IEQ problems. The exclusion criteria are described in detail in Additional file 1: Fig. S1. The complete data were available for 8775 primary school pupils (99 school buildings) and 3410 secondary school pupils (30 school buildings), which formed the analytical samples of the current study. The additional analyses were conducted in parental data for supplementary evidence; the data was available for 3540 parents of primary school pupils (grades 1–6, 88 school buildings).

### Outcome measures

#### Symptom reporting

Symptoms were assessed via the questionnaire developed for secondary school pupils (and parents for primary school pupils) and via the simplified questionnaire for primary school pupils. The questionnaire for secondary school pupils included 18 questions related to pupil’s respiratory symptoms: “Have you had any of the following respiratory symptoms in the last 4 weeks: a) runny nose, b) stuffy nose, c) sore throat, d) hoarseness, e) cough, f) nocturnal cough, g) shortness of breath, h) wheezing?”; and other symptoms: “Have you had any other symptoms in the last 4 weeks: a) itchy eye, b) watery eyes, c) rash, d) itchy skin, e) bleeding from the nose, f) muscle pain, g) joint pain/swelling, h) fatigue, i) headache, j) concentration difficulties?”. The corresponding questions in parental questionnaire were “Has your child had any of the following respiratory symptoms in the last 4 weeks?” and “Has your child had any other symptoms in the last 4 weeks?” with the same list of respiratory and other symptoms. All items had four response options (i.e., 0 = “never”, 1 = “sometimes”, 2 = “every week”, and 3 = “almost every day”). Based on the results of our previous study [35], symptoms were classified into five symptom groups: respiratory (i.e., runny nose, stuffy nose, cough, hoarseness, and sore throat), lower respiratory (i.e., nocturnal cough, shortness of breath, wheezing), eye (i.e., itchy eyes and watery eyes), skin (i.e., itchy skin and rash), and general symptoms (i.e., fatigue, concentration difficulties, and headache). To focus on the more severe symptoms, each symptom item was dichotomized (i.e., 0 = “never or sometimes” and 1 = “every week or almost every day”). A symptom group was coded as “1” if a child reported having at least one of the symptoms included in the symptom group and “0” if a child reported no symptoms.

The questionnaire to primary school pupils comprised 10 questions, and the children were asked whether they experienced the abovementioned symptoms during the last 2 weeks: “Have you had during the last 2 weeks the following: a) stuffy or runny nose, b) sore throat, c) hoarseness, d) cough, e) shortness of breath, f) wheezing, g) itchy eyes or watery eyes, h) itchy skin or rash, i) fatigue, j) headache”. Pictures of a child experiencing the symptom were included in the questionnaire to help primary school pupils better understand the questions. All items had three response options (i.e., 0 = “never”, 1 = “sometimes”, 2 = almost every day”) and were then dichotomized (0 = “never”, 1 = “sometimes or almost every day”). Likewise, five symptom groups were created: respiratory (i.e., runny nose, stuffy nose, cough, hoarseness, and sore throat), lower respiratory (i.e., shortness of breath and wheezing), eye (i.e., itchy eyes and watery eyes), skin (i.e., itchy skin and rash), and general symptoms (i.e., fatigue and headache). A symptom group was again coded as “1″ if a child reported having at least one of the symptoms included in the symptom group and “0″ if a child reported no symptoms.

#### Symptoms related to school environment

All survey respondents were also asked whether they think the symptoms from the five abovementioned symptom groups are especially related to the school environment: “Do you think that some of the symptoms are especially related to the school environment?”. Each symptom group was asked separately, and the question had three response options (0 = “no/no symptoms”, 1 = “yes”, 9 = “I do not know”). In this study, only those respondents who scored positively in the symptom group (i.e., reported having at least one symptom every week or almost every day) and related the corresponding symptom group to school environment were coded as “1” and the rest as “0”. The proportions of primary school pupils who reported having symptoms but did not know whether they were related to school environment were the following: 35% for respiratory symptoms, 14% for lower respiratory symptoms, 21% for eye symptoms, 19% for skin symptoms, and 27% for general symptoms. The corresponding proportions for secondary school pupils were 36, 21, 26, 23, and 28%, respectively.

### Exposure

#### Expert evaluation of indoor environmental quality

Same experts assessed the indoor environmental quality in all school buildings. The assessment was based on all existing data from each school, and no special visits were done. All experts had a long work history with schools at the City of Helsinki. One expert came from the Occupational Safety Section of the City of Helsinki and the two others from the Building Maintenance Section of the City of Helsinki. In the course of several sessions, the experts rated all the school buildings by reaching a consensus concerning the relative rating of the school buildings using a building checklist with the following criteria: a) moisture and mold damage, b) insufficient ventilation, c) unsatisfactory temperature conditions (too cold or too hot), d) building structures with high risk of moisture damage, e) strong smell of mold, f) other strong smells, g) extensive coating damage and emission due to moisture damage in concrete floor structures (most commonly refers to situations where adhesive or plasticizer of a polyvinyl chloride or similar floor reacts with an alkaline moisture of the concrete slab causing volatile organic compounds emissions), h) mineral fibers in building or in the ventilation system, and i) other significant impurities in the ventilation system. Item a) was rated on the scale from 0 = “no damage” to 3 = “extensive damage and significant extent of repair”, while items from b) to i) were rated on the scale 0 = “no”, 1 = “possible”, 2 = “yes”. The experts filled in the building checklist before the questionnaire survey. These criteria for expert evaluation were taken from the report by Finnish Institute of Occupational Health [36], which summarizes a comprehensive system for assessing indoor air problems at workplace, taking into account Finnish legislation and guidelines [37].

We created a summary score of IEQ problems in school buildings by summing up the following highly correlated IEQ indicators: a) moisture and mold damage, b) insufficient ventilation, c) unsatisfactory temperature conditions, d) building structures with high risk of moisture damage, e) strong smell of mold, and f) other strong smells. Such IEQ indicators as damage in concrete floor structures, mineral fibers in building or in the ventilation system, and other significant impurities in the ventilation system were rare (2–4% of all school buildings, Additional file 1: Table S4) and not included to the summary score. To calculate a summary score, we recoded the scale of moisture and mold damage as 0 (no damage), 1 (minor and easily repaired damage), and 2 (substantial or extensive damage), and for the rest of IEQ indicators we used their original scales (0–2). In our data, strong smell of mold and other strong smells scored only from 0 to 1; therefore, the summary score ranged from 0 (no IEQ problems) to 10 (severe IEQ problems).

To validate the expert assessment of IEQ in school buildings, we performed the assessment of moisture and mold damage in a subsample of 43 school buildings by independent inspections and compared the degree of agreement between experts’ and inspectors’ assessments. Two inspectors visited the schools and additionally used previous IEQ and structural condition investigation documents, as well as all the other inspection documents on indoor air measurements. The inspectors used mainly visual, non-intrusive observation, and were blinded to the questionnaire results and the experts’ ratings. Inspectors summarized their assessment of moisture and mold damage using the same grading as the experts. We found a moderate correlation between moisture and mold damage rated by experts and by inspectors (Kendall’s tau = 0.33, *p* = 0.023); whereas the results from the concordance analysis demonstrated substantial agreement between the two ratings (weighted kappa = 69%).

### Confounding variables

Previous research has shown that younger age, female sex, allergic diseases, and tobacco smoking (passive in children and active in adults) are related to higher symptom reporting [7, 9, 27, 38, 39]. We, therefore, controlled for pupils’ age and sex, allergic diseases, which included asthma, hay fever, and atopic rash experienced during the last 12 months (0 = “no”, 1 = “yes”), and smoking to take into account the role of other factors than IEQ related to symptom reporting. We also controlled for attending the Swedish-speaking school (0 = “no”, 1 = “yes”) to adjust for the difference in questionnaires’ languages. Smoking was coded as passive smoking for primary school pupils (0 = “no one smokes”, 1 = “mother, father or another person in the household smokes”) and active smoking for secondary school pupils (0 = “no”, 1 = “yes”).

### Statistical analysis

We first defined the groups of school buildings with similar IEQ problems using the Latent Class Analysis (LCA). LCA models with 1 to 5 classes were fitted to 7 items measuring IEQ problems in 135 school buildings. Variables were entered into the models as binary (0 = “no problem”, 1 = “possible or existing problem”). Criteria used to select the LCA final model [40] included the change in likelihood between models, Bayesian Information Criterion (BIC), Akaike Information Criterion (AIC), and entropy. The percentage change in the log-likelihood for each model was compared by selecting a model with not too much difference when adding another class. BIC and AIC are descriptive goodness-of-fit indices wherein lower values indicate a better model fit. Entropy reflects the classification accuracy of placing observations into latent classes based on their model-based posterior probabilities; it ranges from 0 to 1 with values close to 1 indicating a better fit. After selecting the final LCA model, the posterior probability of belonging to each group was obtained for each school building.

We then used a multilevel logistic regression analysis to examine the associations between IEQ problems (independent variable, assessed on school level) and five symptom scores (dependent variables, assessed on pupil level) in 3–6 grade pupils and 7–9 grade pupils. Given that the data is hierarchical (pupils are nested within schools), a two-level model with school buildings as random intercept was built to account for the dependence among the pupils at the same school. We tested several models for each symptom score and analyzed IEQ problems as a) a summary score and b) latent classes of school buildings. We additionally conducted the analysis with separate IEQ problems (i.e., moisture and mold damage, insufficient ventilation, unsatisfactory temperature conditions, building structures with high risk of moisture damage, smell of mold, and other strong smells). All models were adjusted for the abovementioned covariates, and the results were reported from the fully-adjusted fixed-effects models. We repeated the main analyses using symptoms related to being at school. For supplementary evidence, we repeated the main analysis in parent-administered questionnaires (grades 1–6) using multilevel logistic regression analysis. All analyses were conducted in Stata 15 [41] using *melogit* command for multilevel analyses and *gsem* command for LCA.

To correct for multiple testing, we conducted a Benjamini-Hochberg test to adjust the *p*-values for the False Discovery Rate [42]. We first collected all p-values from the analyses using IEQ summary score and IEQ latent classes as predictors and symptoms reported in relation to school environment and without such relation as outcomes in three samples, ordered them from smallest to largest, and ranked. We then compared each individual p-value to its Benjamini-Hochberg critical value using the False Discovery Rate of 0.10 and 0.05.

## Results

The mean age of 3–6 grade pupils was 10.7 (SD = 1.22), ranged from 7 to 14 years. Half of the pupils were female (51.5%). The most prevalent were respiratory (21.2%) and general symptom groups (20.5%) in 3–6-grade pupils’ reports (Table 1). Among those pupils who reported having respiratory symptoms, 7.1% related them to the school environment, and among those reporting general symptoms, 12.2% related them to the school environment.

**Table 1:** Descriptive statistics

	3–6 grade pupils		7–9 grade pupils	
	(n = 8775)		(*n* = 3410)	
	n	%	n	%
Age (years)	Mean = 10.7, SD = 1.22		Mean = 14.2, SD = 0.95	
7–8 (3–6 grade) / 13 (7–9 grade)	35	0.40	989	29.0
9–10 (3–6 grade) / 14 (7–9 grade)	4079	46.5	1083	31.8
11–12 (3–6 grade) / 15 (7–9 grade)	4182	47.7	1085	31.8
13–14 (3–6 grade) / 16–17 (7–9 grade)	479	2.5	253	7.4
Female sex	4523	51.5	1786	52.4
Asthma	530	6.0	211	6.2
Hay fever	1122	12.8	754	22.1
Atopic rash	852	9.7	427	12.5
Smoking	2271	25.9	934	27.4
Attending Swedish-speaking school	865	9.9	595	17.5
Symptoms^a^				
Respiratory	1861 (619)	21.2 (7.1)	583 (245)	17.1 (7.2)
Lower respiratory	212 (82)	2.4 (0.9)	320 (77)	9.4 (2.3)
Eye	627 (376)	7.2 (4.3)	569 (280)	16.7 (8.2)
Skin	743 (339)	8.5 (3.9)	207 (56)	6.1 (1.6)
General	1800 (1070)	20.5 (12.2)	1974 (1012)	57.9 (29.7)

^a^Symptoms were reported in general, without attribution to the school environment and in relation to the school environment (in parentheses)

The mean age of 7–9 grade pupils was 14.2 (SD = 0.95), ranged from 13 to 17 years. Likewise, half of the pupils were female (52.4%). The most prevalent were general symptoms (57.9%), respiratory symptoms (17.1%), and eye symptoms (16.7%). Among those pupils who reported having general symptoms, 29.7% related them to the school environment. The corresponding percentages for respiratory and eye symptoms were 7.2 and 8.2%.

**Fig. 1:**
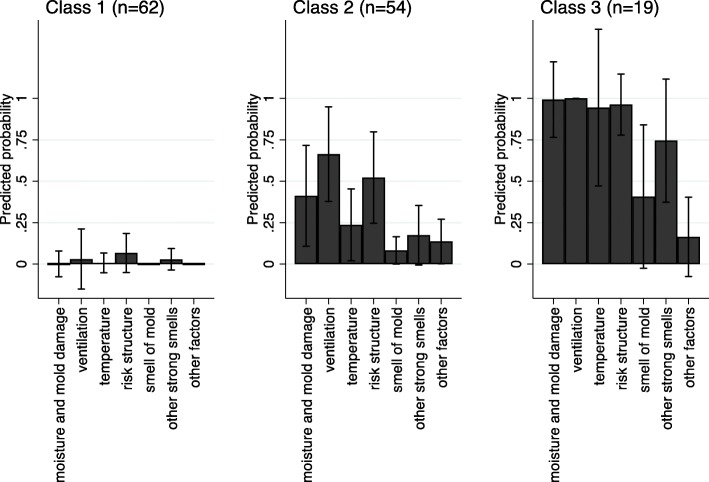
Predicted probabilities for levels of IEQ problems from 3 classes for school buildings (*n* = 135). Class 1: “Good IEQ”, Class 2: “Moderate IEQ”, Class 3: “Poor IEQ”

The most common IEQ problems were insufficient ventilation (44%), building structures with a high risk of moisture damage (38%), and moisture and mold damage (30%) (Additional file 1: Table S2). The rarest IEQ problems were mineral fibers in the ventilation system (4%), other impurities in the ventilation system (4%), and damage in concrete floor structures (2%). The following IEQ problems were highly correlated with each other: moisture and mold damage, insufficient ventilation, unsatisfactory temperature conditions, and building structures with a high risk of moisture damage (r ranged from 0.46 to 0.60, *p* < 0.001; Additional file 1: Table S2). The results from LCA showed that the 2-class model had the best fit in terms of BIC and also entropy was good, but AIC favored the 4-class model (Additional file 1: Table S3). Based on these results and the need to assess dose-response relationships, we decided to use the 3-class solution in the present analyses. The following labels were assigned to the latent classes: a) ‘Good IEQ’ (46%, *n* = 62 (based on most likely class membership)), b) ‘Moderate IEQ’ (40%, *n* = 54), c) ‘Poor IEQ’ (14%, *n* = 19). The probabilities for selected categories of the IEQ indicators within each class are shown in Fig. 1. The latent class of ‘Good IEQ’ corresponds to Mean = 0.16 of the IEQ summary score, ‘Moderate IEQ’ to Mean = 2.4, and ‘Poor IEQ’ to Mean = 7.4.

**Fig. 2:**
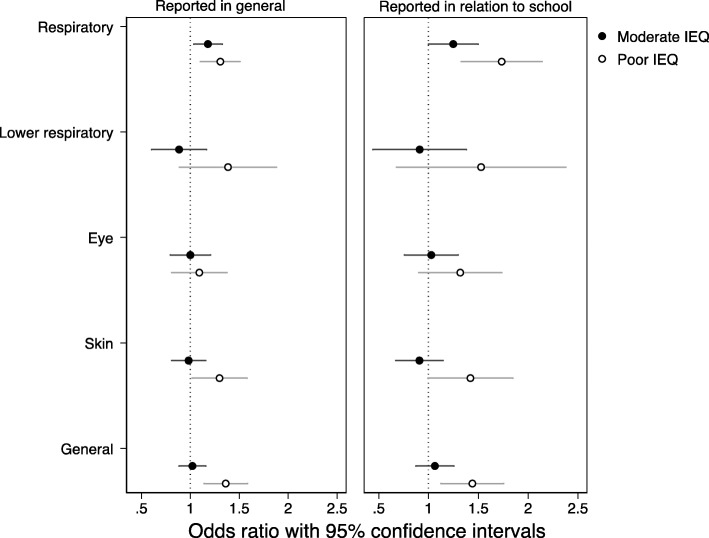
Adjusted odds ratios for the associations between latent classes of IEQ (Good IEQ is the reference) and symptoms reported in general and in relation to the school environment of primary school pupils (*n* = 8775 pupils, 99 school buildings). *Note*. All analyses were adjusted for pupils’ age, sex, asthma, hay fever, atopic rash, parental smoking, and attending Swedish-speaking school. Separate models were tested for each symptom score (odds ratios and 95% CI are listed in Additional file 1: Table S21)

A summary score of IEQ problems in schools was associated with higher reporting of respiratory (OR = 1.04, 95% CI: 1.02, 1.06) and general (OR = 1.03, 95% CI: 1.01, 1.05) symptoms among primary school pupils (Table 2). The associations became stronger with the increasing number of IEQ problems in schools, thus showing a dose-response effect (Moderate IEQ: OR = 1.18, 95% CI: 1.04, 1.34; Poor IEQ: OR = 1.31, 95% CI: 1.12, 1.53; Fig. 2). The analyses with separate IEQ indicators revealed that respiratory symptoms were related to moisture and mold damage, insufficient ventilation, unsatisfactory temperature conditions, building structures with a high risk of moisture damage, and strong smells in school (Additional file 1 Table S4). General symptoms were associated with all abovementioned IEQ indicators except building structures with a high risk of moisture damage (Additional file 1: Table S5). No associations were found between IEQ problems in schools and lower respiratory and eye symptoms (Table 2; Additional file 1: Table S6–S7); whereas higher reporting of skin symptoms was related to schools with poor IEQ, but not moderate IEQ (OR = 1.30, 95% CI: 1.05, 1.61). Moisture and mold damage, insufficient ventilation, unsatisfactory temperature conditions, and mold odor were related to skin symptoms (Additional file 1: Table S8). When symptoms were reported in relation to being in school, all the abovementioned associations were similar in direction but somewhat stronger in magnitude.

**Table 2:** Associations between a summary score of indoor environmental quality problems and different symptom scores of primary and secondary school pupils reported in general and in relation to the school environment

Symptom score	In general^a^		In relation to school^b^	
	OR	95% CI	OR	95% CI
3–6 grade pupils (n = 8775)				
Respiratory	**1.04**	**1.02 to 1.06**	**1.07**	**1.04 to 1.10**
Lower respiratory	1.04	0.99 to 1.09	1.06	0.98 to 1.14
Eye	1.01	0.97 to 1.04	1.02	0.98 to 1.07
Skin	1.03	1.00 to 1.06	1.02	0.98 to 1.07
General	**1.03**	**1.01 to 1.05**	**1.04**	**1.01 to 1.07**
7–9 grade pupils (n = 3410)				
Respiratory	**1.05**	**1.01 to 1.09**	**1.09**	**1.02 to 1.17**
Lower respiratory	1.03	0.99 to 1.07	1.08	0.96 to 1.21
Eye	1.01	0.97 to 1.04	1.03	0.98 to 1.08
Skin	1.01	0.96 to 1.06	**1.10**	**1.01 to 1.19**
General	1.02	0.99 to 1.05	**1.04**	**1.01 to 1.08**

Note. *OR* Odds ratio, *CI* Confidence intervals. ^a^Symptoms reported without attribution to the school environment (in general). ^b^Symptoms reported in relation to the school environment. IEQ summary score (ranged 0–10) is used as a continuous variable

All analyses were adjusted for pupils’ age, sex, asthma, hay fever, atopic rash, parental smoking, and attending Swedish-speaking school. Separate models were tested for each symptom score. Results highlighted in bold are statistically significant at *p*<0.05

**Fig. 3:**
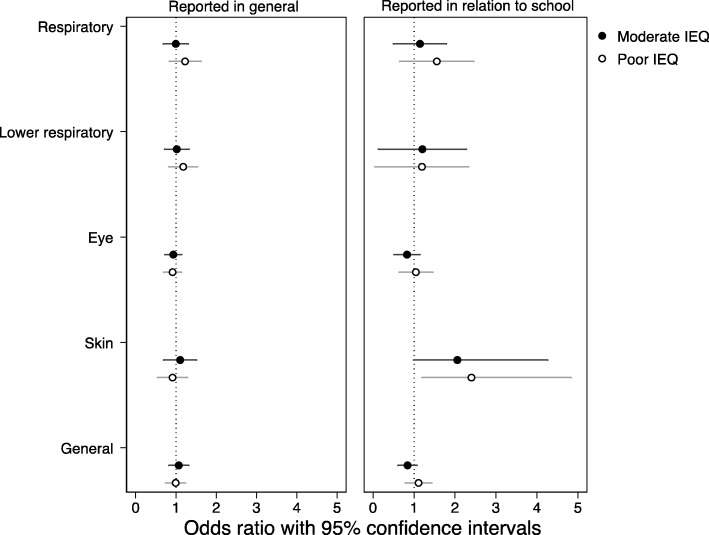
Adjusted odds ratios for the associations between latent classes of IEQ (Good IEQ is the reference) and symptoms reported in general and in relation to the school environment of secondary school pupils (*n* = 3410 pupils, 30 school buildings). *Note*. All analyses were adjusted for pupils’ age, sex, asthma, hay fever, atopic rash, parental smoking, and attending Swedish-speaking school. Separate models were tested for each symptom score (odds ratios and 95% CI are listed in Additional file 1: Table S22)

IEQ problems were associated with respiratory symptoms among secondary school pupils (OR = 1.05, 95% CI: 1.01, 1.09; Table 2). The association was observed only with a summary score of IEQ problems, but not with latent classes of IEQ in schools (Fig. 3). No associations were found between IEQ summary score or latent classes and all other symptoms. The analyses with separate IEQ indicators showed that respiratory symptoms were associated with moisture and mold damage, unsatisfactory temperature conditions, and building structures with a high risk of moisture damage (Additional file 1 Table S9). The associations between separate IEQ indicators were also found for lower respiratory and general symptoms. Lower respiratory symptoms were related to moisture and mold damage and unsatisfactory temperature conditions (Additional file 1: Table S11), whereas general symptoms were associated with moisture and mold damage and building structures with a high risk of moisture damage (Additional file 1: Table S10). No associations were found between separate IEQ indicators and eye or skin symptoms (Additional file 1: Table S12 and S13). Likewise, when symptoms were reported in relation to being in school, all the above mentioned associations were similar in direction but somewhat stronger in magnitude. In addition, the associations were found between IEQ summary score and skin (OR = 1.10, 95% CI: 1.01, 1.19) and general symptoms (OR = 1.04, 95% CI: 1.01, 1.08).

No significant associations were found between the rarest IEQ factors (i.e., extensive coating damage and emission due to moisture damage in concrete floor structures, mineral fibers in building or in the ventilation system, and other significant impurities in the ventilation system) and symptom reporting both in primary and secondary school pupils (results not shown).

We repeated the main analyses in the parental dataset to provide supplementary evidence for our research questions. Summary score of IEQ problems was associated with higher reporting of respiratory (1.07, 95% CI: 1.03, 1.11), lower respiratory (1.05, 95% CI: 1.01, 1.10), and general (1.04, 95% CI: 1.01, 1.07) symptoms in questionnaires filled in by parents for their primary school pupils (Additional file 1: Table S14). Similar to results in primary school pupils’ dataset, the analyses with separate IEQ indicators revealed that respiratory symptoms were related to moisture and mold damage, insufficient ventilation, unsatisfactory temperature conditions, building structures with a high risk of moisture damage, and strong smells in school (Additional file 1: Table S15). The associations for the rest of the symptoms and separate IEQ indicators are shown in Additional file 1: Table S16–19. We also found that the associations between IEQ and symptoms reported in relation to being in school were stronger compared to associations when symptoms were reported without relation to the school environment (in general).

With 5 symptoms reported in general and 5 symptoms reported in relation to the school environment and 3 predictors (i.e., summary score of IEQ and latent classes of IEQ – Moderate IEQ and Poor IEQ (Good IEQ used as a reference)), we conducted 30 tests in each sample resulting in 90 tests in total. Of the 34 statistically significant associations, all would have been statistically significant when allowing a false discovery rate of 0.10, suggesting that these associations were unlikely to be false positives. However, when controlling for a false discovery rate of 0.05, only 16 associations would have been statistically significant (Additional file 1: Table S20).

## Discussion

This study examined associations between IEQ problems in schools and symptom reporting of pupils, and whether associations became stronger if participants relate symptoms to the school environment. We found the associations between the summary score of IEQ problems and higher reporting of respiratory and general symptoms among both primary and secondary school pupils. We also observed the dose-response associations between latent classes of IEQ problems in schools (i.e., Good IEQ, Moderate IEQ, and Poor IEQ) and symptom reporting. Some associations were also observed between IEQ indicators and lower respiratory as well as skin symptoms, but not eye symptoms.

We further tested whether the associations between school IEQ and symptom reporting differ when symptoms are asked in relation to being in school and when symptoms are asked without relation to the school environment (i.e., experienced in general). We found that the associations between IEQ and symptoms related to the school environment were somewhat stronger in magnitude compared to associations with symptoms reported without such relation in both reports of primary and secondary school pupils. To the best of our knowledge, these associations have not been tested previously. It should be noted, however, that prevalence of respondents who reported symptoms but do not know whether they are related to school environment was quite large in all samples (e.g., 35–36% for respiratory symptoms and 27–28% for general symptoms).

In line with previous studies [13–18], we found the strongest associations between IEQ and respiratory symptoms among pupils in primary and secondary schools. We also observed the associations between IEQ and general symptoms (i.e., tiredness, headache, and difficulties concentrating), whereas previous studies conducted in schools showed no such associations [18, 22] or small and nearly significant associations [23]. To continue, we found some associations between separate IEQ indicators (e.g., moisture and mold damage) and lower respiratory symptoms, which accords with previous studies [13]. We also found the associations between poor IEQ (as well as several IEQ indicators) and skin symptoms. Finally, no associations were found regarding eye symptoms in primary and secondary school pupils’ reports.

Among separate IEQ indicators, especially moisture and mold damage, unsatisfactory temperature conditions and presence of building structures with a high risk of moisture damage were related to higher reports of respiratory symptoms in primary and secondary school pupils, which is in line with previous studies [13, 14, 16–18]. We also found that insufficient ventilation was related to higher reporting of respiratory symptoms but only among primary school pupils. Previous studies have also shown that insufficient ventilation, which often does not correspond even to the minimum rates of ventilation standard, is common in schools and is related to increased respiratory symptoms and illness absence of pupils [1, 18, 22, 43, 44]. Regarding general symptoms, one study [23] has found marginally significant associations between general symptoms and temperature conditions. However, we found that not only improper temperature conditions, but also insufficient ventilation and mold odor were related to increased reports of general symptoms in primary school pupils’ reports. No associations were found regarding eye symptoms in primary and secondary school pupils’ reports. In contrast, skin symptoms were related to temperature, ventilation, and mold odor in primary school pupils’ reports, but not in secondary school pupils’ reports.

One noticeable finding of this study was the very low response rate (below 20%) of parents of primary school pupils in this questionnaire survey. This suggests that some new incentive strategies are required during survey data collection to achieve adequate response rate among parents of primary school pupils. This is especially relevant for studies like the present one, in which not only schools with suspected problems are surveyed, but all schools in the given area are included in the analyses. One possibility is to use pupil-administered questionnaires in primary schools, given that they are easier to administer to pupils in schools than to their parents, which yields clearly higher response rates, as evidenced in this study. After conducting additional analysis in parental data, we found that the associations between IEQ problems and symptoms reported in general (without relation to school environment) did not differ between questionnaires filled in by parents or their children. When symptoms were reported in relation to the school environment, the associations between IEQ problems and symptoms were much stronger in parental questionnaires than in pupils’ reports. However, due to the low response rate of parents and possible self-selection bias [45], these results should be interpreted with caution.

This study has several limitations which should be taken into account when interpreting the results. First, indoor environmental quality is a complex and multifaceted concept, and its identification and evaluation include many challenges and uncertainties [37, 46]. In our study, we did not conduct any specific measures (e.g., CO2 levels, oxides of sulfur and nitrogen) nor special visits to assess the IEQ of each building but relied on consensus assessment of experts with good knowledge of the school buildings under study. The experts provided a relative ranking of the buildings using the given criteria without explicit reference to predefined cut points. The advantage compared to some earlier large-scale studies [18] is that all buildings were rated by the same experts, although special visits with the standardized protocol would be optimal [13]. The assessment method used in the present study may partly explain the high correlations between different IEQ indicators. The experts’ assessment may have also been influenced by previous reports of symptoms related to poor indoor air quality in the schools, although the assessment was done before the questionnaire survey. To address these issues, we have performed independent inspectors’ visits to a subsample of 43 school buildings to assess moisture and mold damage. Inspectors’ assessment of moisture and mold damage was based on the available information from the previous condition investigation reports and a single assessment visit with mainly visual and non-intrusive observations, which has its own drawbacks. Nevertheless, we examined the associations between experts’ ratings and inspectors’ assessment of moisture and mold damage and found a moderate positive association between these two assessments.

Given that the assessment of moisture and mold damage has proven difficult [47], previous studies have shown only moderate agreement between inspectors, even when they used the same detailed protocol in homes [48]. The classification of small damage is especially problematic in larger buildings, such as schools. This was also evident in the present study: the independent inspectors rated minor and easily repaired moisture damage as 1 on the scale from 0 (no damage) to 3 (extensive damage and significant extent of repair); whereas the experts rated such minor damage as 0 given that it did not have a probable impact on the IEQ of the whole building. Nevertheless, we tested the degree of concordance between these two ratings and found a substantial agreement between them suggesting that experts’ and inspectors’ ratings correspond more or less closely. Also, the fact that the assessments were done at the school building level, but not at the classroom level, may lead to measurement imprecision, as especially primary school pupils spend most of their time in the same classes. There were also some differences in the questionnaires developed for primary school pupils and for secondary school pupils (as well as parents). Primary school pupils’ questionnaires comprised fewer questions on symptoms, questions had one less response category, and the time period used was different, as compared to secondary school pupils’ questionnaires. We also cannot eliminate the possibility of self-selection bias since the response rate was on average 50% in among both primary and secondary school pupils and no information was available on non-responders. Previous studies have found that children with higher family socioeconomic status, a greater number of health symptoms, and having non-smoking parents are more likely to participate in a study [45]; therefore, it is possible that our sample may also overrepresent pupils who have more symptoms. The response rates in the pupil-administered questionnaires were, however, clearly higher than in the questionnaires administered to the parents (below 20%). Lastly, this study is based solely on health self-reports, and no objective measurements of pupils’ health were available.

Finally, the focus of this study was on physical, but not psychosocial, characteristics of the school environment and symptom reporting, although both characteristics are important in indoor air research [46, 49]. Previous studies have shown that parents who are worried about school IEQ report more symptoms for their children than non-worried parents [28], and that socioemotional difficulties of pupils are associated with more indoor air-related symptoms [50]. It is possible, therefore, that especially those pupils who related their symptoms to the school environment could be more worried, less satisfied with their school environment, and have problems in teacher-pupil relations or other socioemotional problems.

## Conclusions

In conclusion, this study shows the relation between expert-assessed indoor environmental quality problems in schools and increased reporting of especially respiratory and general symptoms in primary and secondary school pupils’ reports. Some associations were also observed with lower respiratory and skin symptoms, but not with eye symptoms. These associations became stronger with the increasing number of IEQ problems in schools, thus showing dose-response effects. Finally, the associations between IEQ and symptoms related to the school environment were somewhat stronger in magnitude compared to associations with symptoms reported without such relation.

## Supplementary information

**Additional file 1: Table S1.** Response rate of parents and pupils in questionnaire survey. **Table S2.** Prevalence estimates and rank correlation matrix between IEQ indicators. **Table S3.** Model fit statistics for latent class analysis models with 1 to 5 classes. **Table S4**. Separate IEQ indicators and respiratory symptoms: primary school pupils. **Table S5.** Separate IEQ indicators and general symptoms: primary school pupils. **Table S6.** Seaparate IEQ indicators and lower respiratory symptoms: primary school pupils. **Table S7.** Separate IEQ indicators and eye symptoms: primary school pupils. **Table S8.** Separate IEQ indicators and skin symptoms: primary school pupils. **Table S9.** Separate IEQ indicators and respiratory symptoms: secondary school pupils. **Table S10.** Separate IEQ indicators and general symptoms: secondary school pupils. **Table S11.** Separate IEQ indicators and lower respiratory symptoms: secondary school pupils. **Table S12.** Separate IEQ indicators and eye symptoms: secondary school pupils. **Table S13.** Separate IEQ indicators and skin symptoms: secondary school pupils. **Table S14.** Associations between summary score and latent classes of IEQ and symptoms: parents of primary school pupils. **Table S15.** Separate IEQ indicators and respiratory symptoms: parents of primary school pupils. **Table S16.** Separate IEQ indicators and general symptoms: parents of primary school pupils. **Table S17.** Separate IEQ indicators and lower respiratory symptoms: parents of primary school pupils. **Table S18.** Separate IEQ indicators and eye symptoms: parents of primary school pupils. **Table S19**. Separate IEQ indicators and skin symptoms: parents of primary school pupils. **Table S20.** Benjamini-Hochberg test of false discovery rate. **Table S21.** Odds ratios and 95% confidence intervals for associations between IEQ and symptoms of primary school pupils. **Table S22.** Odds ratios and 95% confidence intervals for the associations between IEQ and symptoms of secondary school pupils. **Figure S1.** Flowchart describing the selection of school buildings and participants.

## Data Availability

The datasets analysed during the current study are not publicly available due to reasons of confidentiality, but are available from the corresponding author on reasonable request, after approval by the data protection authorities.

## Abbreviations

AIC: Akaike Information Criterion
BIC: Bayesian Information Criterion
CI: Confidence interval
IEQ: Indoor environmental quality
LCA: Latent class analysis
OR: Odds ratio
SD: Standard deviation
