# Supplementary material for: Associations between indoor environmental quality in schools and symptom reporting in pupil-administered questionnaires
Source: Environ Health. 2019 Dec 27;18:115. doi: 10.1186/s12940-019-0555-6 (PMC6935098; doi:10.1186/s12940-019-0555-6)
Supplement: Supplementary file 1 — Additional file 1: Table S1. Response rate of parents and pupils in questionnaire survey. Table S2. Prevalence estimates and rank correlation matrix between IEQ indicators. Table S3. Model fit statistics for latent class analysis models with 1 to 5 classes. Table S4. Separate IEQ indicators and respiratory symptoms: primary school pupils. Table S5. Separate IEQ indicators and general symptoms: primary school pupils. Table S6. Seaparate IEQ indicators and lower respiratory symptoms: primary school pupils. Table S7. Separate IEQ indicators and eye symptoms: primary school pupils. Table S8. Separate IEQ indicators and skin symptoms: primary school pupils. Table S9. Separate IEQ indicators and respiratory symptoms: secondary school pupils. Table S10. Separate IEQ indicators and general symptoms: secondary school pupils. Table S11. Separate IEQ indicators and lower respiratory symptoms: secondary school pupils. Table S12. Separate IEQ indicators and eye symptoms: secondary school pupils. Table S13. Separate IEQ indicators and skin symptoms: secondary school pupils. Table S14. Associations between summary score and latent classes of IEQ and symptoms: parents of primary school pupils. Table S15. Separate IEQ indicators and respiratory symptoms: parents of primary school pupils. Table S16. Separate IEQ indicators and general symptoms: parents of primary school pupils. Table S17. Separate IEQ indicators and lower respiratory symptoms: parents of primary school pupils. Table S18. Separate IEQ indicators and eye symptoms: parents of primary school pupils. Table S19. Separate IEQ indicators and skin symptoms: parents of primary school pupils. Table S20. Benjamini-Hochberg test of false discovery rate. Table S21. Odds ratios and 95% confidence intervals for associations between IEQ and symptoms of primary school pupils. Table S22. Odds ratios and 95% confidence intervals for the associations between IEQ and symptoms of secondary school pupils. Figure S1. Flowchart describing the [file 12940_2019_555_MOESM1_ESM.docx]

**Associations between indoor environmental quality in schools and symptom reporting in pupils-administered questionnaires**

Additional file 1. Supplementary Materials

| Table S1. | Response rate of parents and pupils in questionnaire survey |
| --- | --- |
| Table S2. | Prevalence estimates and rank correlation matrix between IEQ indicators (n=135 school buildings) |
| Table S3. | Model fit statistics for latent class analysis models with 1 to 5 classes |
| Table S4. | Adjusted odds ratios for the associations between IEQ indicators and respiratory symptoms reported by primary school pupils (n = 8775 pupils, 99 school buildings) |
| Table S5. | Adjusted odds ratios for the associations between IEQ indicators and general symptoms reported by primary school pupils (n = 8775 pupils, 99 school buildings) |
| Table S6. | Adjusted odds ratios for the associations between IEQ indicators and lower respiratory symptoms reported by primary school pupils (n = 8775 pupils, 99 school buildings) |
| Table S7. | Adjusted odds ratios for the associations between IEQ indicators and eye symptoms reported by primary school pupils (n = 8775 pupils, 99 school buildings) |
| Table S8. | Adjusted odds ratios for the associations between IEQ indicators and skin symptoms reported by primary school pupils (n = 8775 pupils, 99 school buildings) |
| Table S9. | Adjusted odds ratios for the associations between IEQ indicators and respiratory symptoms reported by secondary school pupils (n = 3410 pupils, 30 school buildings) |
| Table S10. | Adjusted odds ratios for the associations between IEQ indicators and general symptoms reported by secondary school pupils (n = 3410 pupils, 30 school buildings) |
| Table S11. | Adjusted odds ratios for the associations between IEQ indicators and lower respiratory symptoms reported by secondary school pupils (n = 3410 pupils, 30 school buildings) |
| Table S12. | Adjusted odds ratios for the associations between IEQ indicators and eye symptoms reported by secondary school pupils (n = 3410 pupils, 30 school buildings) |
| Table S13. | Adjusted odds ratios for the associations between IEQ indicators and skin symptoms reported by secondary school pupils (n = 3410 pupils, 30 school buildings) |
| Table S14. | Adjusted odds ratios for the associations between indoor environmental quality (IEQ) and symptoms reported by parents for their primary school pupils (n = 3540 pupils, 88 school buildings) |
| Table S15. | Adjusted odds ratios for the associations between IEQ indicators and respiratory symptoms reported by parents for their primary school pupils (n = 3540 parents, 88 school buildings) |
| Table S16. | Adjusted odds ratios for the associations between IEQ indicators and general symptoms reported by parents for their primary school pupils (n = 3540 parents, 88 school buildings) |
| Table S17. | Adjusted odds ratios for the associations between IEQ indicators and lower respiratory symptoms reported by parents for their primary school pupils (n = 3540 parents, 88 school buildings) |
| Table S18. | Adjusted odds ratios for the associations between IEQ indicators and eye symptoms reported by parents for their primary school pupils (n = 3540 parents, 88 school buildings) |
| Table S19. | Adjusted odds ratios for the associations between IEQ indicators and skin symptoms reported by parents for their primary school pupils (n = 3540 parents, 88 school buildings) |
| Table S20. | Benjamini-Hochberg test of false discovery rate |
| Table S21. | The odds ratios and 95% confidence intervals for the associations between indoor environmental quality (IEQ) and symptoms of primary school pupils (n=8775, 99 school-buildings). |
| Table S22. | The odds ratios and 95% confidence intervals for the associations between indoor environmental quality (IEQ) and symptoms of primary school pupils (n=3410, 30 school-buildings). |
| Figure S1. | Flowchart describing how the school buildings and participants in three samples were selected for the study. |

**Table S1. Response rate of parents and pupils in questionnaire survey**

|  | Questionnaire completion | | | | |
| --- | --- | --- | --- | --- | --- |
|  | 2017 | |  | 2018 | |
| Grade level | Parents | Pupils |  | Parents | Pupils |
| 1-2 | n = 2426 (20%) | NA |  | n = 1513 (13%) | NA |
| 3-6 |  | n = 5360 (68%) |  |  | n = 4475 (57%) |
| 7-9 | NA | n = 1905 (55%) |  | NA | n = 2059 (48%) |

Parents completed the questionnaires for their 1-6-grade pupils.

3-6 grade pupils (primary school) and 7-9 grade pupils (secondary school) completed the questionnaires themselves.

**Table S2. Prevalence estimates and rank correlation matrix between IEQ indicators (n=135 school buildings)**

|  |  | 1. | 2. | 3. | 4. | 5. | 6. | 7. | 8. | 9. |
| --- | --- | --- | --- | --- | --- | --- | --- | --- | --- | --- |
| 1. | Moisture and mold damage | 1 |  |  |  |  |  |  |  |  |
| 2. | Ventilation | **0.58** | 1 |  |  |  |  |  |  |  |
| 3. | Temperature | **0.46** | **0.57** | 1 |  |  |  |  |  |  |
| 4. | Building risk structures | **0.60** | **0.56** | **0.47** | 1 |  |  |  |  |  |
| 5. | Smell of mold | **0.39** | **0.29** | **0.28** | **0.21** | 1 |  |  |  |  |
| 6. | Other strong smells | **0.33** | **0.52** | **0.46** | **0.28** | **0.25** | 1 |  |  |  |
| 7. | Damage in concrete floor structures | 0.09 | 0.16 | 0.03 | 0.05 | -0.05 | 0.06 | 1 |  |  |
| 8. | Mineral fibers in ventilation system | 0.12 | **0.18** | -0.02 | 0.04 | -0.06 | 0.01 | **0.24** | 1 |  |
| 9. | Other impurities in ventilation system | 0.13 | 0.09 | 0.07 | 0.15 | **0.21** | **0.21** | 0.24 | -0.03 | 1 |
|  | *Prevalence estimates, n (%)* |  |  |  |  |  |  |  |  |  |
|  | 0 | 93 (70%) | 76 (56%) | 104 (77%) | 83 (62%) | 123 (91%) | 110 (81%) | 132 (98%) | 130 (96%) | 129 (96%) |
|  | 1 | 28 (20%) | 35 (26%) | 23 (17%) | 31 (23%) | 12 (9%) | 25 (19%) | 3 (2%) | 5 (4%) | 5 (4%) |
|  | 2 | 14 (10%) | 24 (18%) | 8 (6%) | 21 (15%) | NA | NA | NA | NA | NA |

Values in bold are statistically significant at the 5% level or lower

**Table S3. Model fit statistics for latent class analysis models with 1 to 5 classes**

| Number of classes | Number of school buildings | Log-likelihood | df | % reduction in L | BIC | AIC | Entropy |
| --- | --- | --- | --- | --- | --- | --- | --- |
| 1 | 135 | -482.2276 | 7 | 0 | 998.7921 | 978.4552 | N/A |
| 2 | 135 | -404.0089 | 15 | 16.2 | 881.597 | 838.0178 | 0.86 |
| 3 | 135 | -392.1648 | 21 | 18.7 | 887.3404 | 826.3296 | 0.71 |
| 4 | 135 | -384.5482 | 27 | 20.3 | 901.5387 | 823.0963 | 0.87 |
| 5 | 135 | -380.6514 | 36 | 21.1 | 937.3027 | 833.3027 | 0.88 |

*Note*. df = degree of freedom, BIC = Bayesian information criterion, AIC = Akaike information criterion.

**Table S4. Adjusted odds ratios for the associations between IEQ indicators and respiratory symptoms reported by primary school pupils (n = 8775 pupils, 99 school buildings)**

|  | Respiratory symptoms | | | | | |
| --- | --- | --- | --- | --- | --- | --- |
|  | In generalᵃ | | | In relation to schoolᵇ | | |
| IEQ problems | n (%)* | OR | 95% CI | n (%)* | OR | 95% CI |
| *Moisture and mold damage* |  |  |  |  |  |  |
| 0 | 1181 (21) | 1 |  | 369 (6) | 1 |  |
| 1 | 412 (21) | 1.04 | 0.90 to 1.21 | 149 (8) | 1.20 | 0.95 to 1.51 |
| 2 | 268 (24) | **1.25** | **1.04 to 1.50** | 101 (9) | **1.42** | **1.07 to 1.89** |
| P-value for trend |  | **0.026** |  |  | **0.009** |  |
|  |  |  |  |  |  |  |
| *Ventilation* |  |  |  |  |  |  |
| 0 | 1015 (20) | 1 |  | 306 (6) | 1 |  |
| 1 | 490 (23) | **1.20** | **1.05 to 1.38** | 172 (8) | **1.37** | **1.11 to 1.71** |
| 2 | 356 (23) | **1.24** | **1.07 to 1.45** | 141 (9) | **1.57** | **1.24 to 1.98** |
| P-value for trend |  | **0.001** |  |  | **0.001** |  |
|  |  |  |  |  |  |  |
| *Temperature* |  |  |  |  |  |  |
| 0 | 1393 (21) | 1 |  | 431 (6) | 1 |  |
| 1 | 332 (23) | **1.18** | **1.01 to 1.39** | 143 (10) | **1.65** | **1.32 to 2.06** |
| 2 | 136 (23) | 1.17 | 0.93 to 1.47 | 45 (8) | 1.20 | 0.85 to 1.69 |
| P-value for trend |  | **0.037** |  |  | **0.004** |  |
|  |  |  |  |  |  |  |
| *Building risk structures* |  |  |  |  |  |  |
| 0 | 1048 (20) | 1 |  | 320 (6) | 1 |  |
| 1 | 468 (23) | **1.22** | **1.06 to 1.40** | 169 (8) | **1.40** | **1.11 to 1.75** |
| 2 | 345 (24) | **1.33** | **1.14 to 1.55** | 130 (9) | **1.55** | **1.21 to 1.99** |
| P-value for trend |  | **0.001** |  |  | **0.001** |  |
|  |  |  |  |  |  |  |
| *Smell of mold* |  |  |  |  |  |  |
| 0 | 1665 (21) | 1 |  | 544 (7) | 1 |  |
| 1 | 196 (23) | 1.09 | 0.89 to 1.34 | 75 (9) | 1.24 | 0.90 to 1.70 |
| P-value |  | 0.392 |  |  | 0.188 |  |
|  |  |  |  |  |  |  |
| *Other strong smell* |  |  |  |  |  |  |
| 0 | 1466 (21) | 1 |  | 463 (7) | 1 |  |
| 1 | 196 (23) | **1.19** | **1.02 to 1.38** | 156 (9) | **1.43** | **1.14 to 1.79** |
| P-value |  | **0.024** |  |  | **0.002** |  |

*Note*. OR = Odds ratio, CI = Confidence intervals

ᵃ Symptoms reported without attribution to school environment (in general)

ᵇ Symptoms reported in relation to school environment

* Number (percentage) of pupils experiencing symptoms.

IEQ problems were analyzed in separate models. All analyses were adjusted for pupils’ age, sex, asthma, hay fever, atopic rash, parental smoking, and attending Swedish-speaking school.

**Table S5. Adjusted odds ratios for the associations between IEQ indicators and general symptoms reported by primary school pupils (n = 8775 pupils, 99 school buildings)**

|  | General symptoms | | | | | |
| --- | --- | --- | --- | --- | --- | --- |
|  | In generalᵃ | | | In relation to schoolᵇ | | |
| IEQ problems | n (%)* | OR | 95% CI | n (%)* | OR | 95% CI |
| *Moisture and mold damage* |  |  |  |  |  |  |
| 0 | 1119 (19) | 1 |  | 644 (11) | 1 |  |
| 1 | 431 (22) | **1.18** | **1.01 to 1.37** | 271 (14) | **1.26** | **1.04 to 1.54** |
| 2 | 250 (23) | 1.18 | 0.97 to 1.43 | 155 (14) | 1.22 | 0.95 to 1.56 |
| P-value for trend |  | **0.029** |  |  | **0.031** |  |
|  |  |  |  |  |  |  |
| *Ventilation* |  |  |  |  |  |  |
| 0 | 991 (19) | 1 |  | 583 (11) | 1 |  |
| 1 | 451 (21) | 1.11 | 0.95 to 1.29 | 266 (12) | 1.09 | 0.89 to 1.33 |
| 2 | 358 (24) | **1.24** | **1.05 to 1.47** | 221 (15) | 1.24 | 0.99 to 1.55 |
| P-value for trend |  | **0.010** |  |  | 0.057 |  |
|  |  |  |  |  |  |  |
| *Temperature* |  |  |  |  |  |  |
| 0 | 1338 (20) | 1 |  | 785 (12) | 1 |  |
| 1 | 351 (25) | **1.32** | **1.12 to 1.55** | 224 (16) | **1.41** | **1.14 to 1.74** |
| 2 | 111 (19) | 0.93 | 0.73 to 1.19 | 61 (10) | 0.86 | 0.62 to 1.19 |
| P-value for trend |  | 0.252 |  |  | 0.372 |  |
|  |  |  |  |  |  |  |
| *Building risk structures* |  |  |  |  |  |  |
| 0 | 1055 (20) | 1 |  | 609 (11) | 1 |  |
| 1 | 438 (21) | 1.09 | 0.92 to 1.28 | 271 (13) | 1.15 | 0.93 to 1.42 |
| 2 | 307 (22) | 1.11 | 0.92 to 1.33 | 190 (13) | 1.18 | 0.93 to 1.50 |
| P-value for trend |  | 0.199 |  |  | 0.111 |  |
|  |  |  |  |  |  |  |
| *Smell of mold* |  |  |  |  |  |  |
| 0 | 1584 (20) | 1 |  | 925 (12) | 1 |  |
| 1 | 216 (26) | **1.26** | **1.03 to 1.55** | 145 (17) | **1.44** | **1.12 to 1.85** |
| P-value |  | **0.025** |  |  | **0.005** |  |
|  |  |  |  |  |  |  |
| *Other strong smell* |  |  |  |  |  |  |
| 0 | 1408 (20) | 1 |  | 825 (12) | 1 |  |
| 1 | 392 (23) | **1.19** | **1.02 to 1.39** | 245 (14) | 1.22 | 0.99 to 1.50 |
| P-value |  | **0.030** |  |  | 0.059 |  |

*Note*. OR = Odds ratio, CI = Confidence intervals

ᵃ Symptoms reported without attribution to school environment (in general)

ᵇ Symptoms reported in relation to school environment

* Number (percentage) of pupils experiencing symptoms.

IEQ problems were analyzed in separate models. All analyses were adjusted for pupils’ age, sex, asthma, hay fever, atopic rash, parental smoking, and attending Swedish-speaking school.

**Table S6. Adjusted odds ratios for the associations between IEQ indicators and lower respiratory symptoms reported by primary school pupils (n = 8775 pupils, 99 school buildings)**

|  | Lower respiratory symptoms | | | | | |
| --- | --- | --- | --- | --- | --- | --- |
|  | In generalᵃ | | | In relation to schoolᵇ | | |
| IEQ problems | n (%)* | OR | 95% CI | n (%)* | OR | 95% CI |
| *Moisture and mold damage* |  |  |  |  |  |  |
| 0 | 135 (2) | 1 |  | 50 (0.9) | 1 |  |
| 1 | 48 (2) | 1.07 | 0.76 to 1.51 | 18 (0.9) | 1.06 | 0.61 to 1.83 |
| 2 | 29 (3) | 1.20 | 0.78 to 1.84 | 14 (1.3) | 1.58 | 0.86 to 2.91 |
| P-value for trend |  | 0.404 |  |  | 0.191 |  |
|  |  |  |  |  |  |  |
| *Ventilation* |  |  |  |  |  |  |
| 0 | 117 (2) | 1 |  | 45 (0.9) | 1 |  |
| 1 | 53 (2) | 1.14 | 0.82 to 1.60 | 19 (0.9) | 1.08 | 0.63 to 1.87 |
| 2 | 42 (3) | 1.25 | 0.86 to 1.81 | 18 (1.2) | 1.37 | 0.78 to 2.39 |
| P-value for trend |  | 0.208 |  |  | 0.294 |  |
|  |  |  |  |  |  |  |
| *Temperature* |  |  |  |  |  |  |
| 0 | 156 (2) | 1 |  | 56 (0.8) | 1 |  |
| 1 | 39 (3) | 1.24 | 0.86 to 1.80 | 19 (1.3) | 1.71 | 1.00 tp 2.90 |
| 2 | 17 (3) | 1.25 | 0.74 to 2.12 | 7 (1.2) | 1.40 | 0.63 to 3.11 |
| P-value for trend |  | 0.214 |  |  | 0.101 |  |
|  |  |  |  |  |  |  |
| *Risk structure* |  |  |  |  |  |  |
| 0 | 120 (2) | 1 |  | 43 (0.8) | 1 |  |
| 1 | 54 (3) | 1.21 | 0.86 to 1.71 | 26 (1.3) | 1.63 | 0.99 to 2.68 |
| 2 | 38 (3) | 1.25 | 0.85 to 1.85 | 13 (0.9) | 1.17 | 0.62 to 2.21 |
| P-value for trend |  | 0.181 |  |  | 0.295 |  |
|  |  |  |  |  |  |  |
| *Smell of mold* |  |  |  |  |  |  |
| 0 | 187 (2) | 1 |  | 70 (0.9) | 1 |  |
| 1 | 25 (3) | 1.15 | 0.73 to 1.81 | 12 (1.4) | 1.38 | 0.74 to 2.59 |
| P-value |  | 0.538 |  |  | 0.315 |  |
|  |  |  |  |  |  |  |
| *Other strong smell* |  |  |  |  |  |  |
| 0 | 169 (2) | 1 |  | 65 (0.9) | 1 |  |
| 1 | 43 (3) | 1.14 | 0.80 to 1.64 | 17 (1) | 1.21 | 0.70 to 2.09 |
| P-value |  | 0.474 |  |  | 0.501 |  |

*Note*. OR = Odds ratio, CI = Confidence intervals

ᵃ Symptoms reported without attribution to school environment (in general)

ᵇ Symptoms reported in relation to school environment

* Number (percentage) of pupils experiencing symptoms.

IEQ problems were analyzed in separate models. All analyses were adjusted for pupils’ age, sex, asthma, hay fever, atopic rash, parental smoking, and attending Swedish-speaking school.

**Table S7. Adjusted odds ratios for the associations between IEQ indicators and eye symptoms reported by primary school pupils (n = 8775 pupils, 99 school buildings)**

|  | Eye symptoms | | | | | |
| --- | --- | --- | --- | --- | --- | --- |
|  | In generalᵃ | | | In relation to schoolᵇ | | |
| IEQ problems | n (%)* | OR | 95% CI | n (%)* | OR | 95% CI |
| *Moisture and mold damage* |  |  |  |  |  |  |
| 0 | 406 (7) | 1 |  | 232 (4) | 1 |  |
| 1 | 133 (7) | 0.93 | 0.74 to 1.18 | 88 (5) | 1.00 | 0.81 to 1.44 |
| 2 | 88 (8) | 1.16 | 0.87 to 1.53 | 56 (5) | 1.26 | 0.89 to 1.78 |
| P-value for trend |  | 0.537 |  |  | 0.198 |  |
|  |  |  |  |  |  |  |
| *Ventilation* |  |  |  |  |  |  |
| 0 | 366 (7) | 1 |  | 212 (4) | 1 |  |
| 1 | 151 (7) | 1.00 | 0.80 to 1.25 | 88 (4) | 1.01 | 0.75 to 1.34 |
| 2 | 110 (7) | 1.01 | 0.79 to 1.31 | 76 (5) | 1.22 | 0.89 to 1.66 |
| P-value for trend |  | 0.922 |  |  | 0.271 |  |
|  |  |  |  |  |  |  |
| *Temperature* |  |  |  |  |  |  |
| 0 | 472 (7) | 1 |  | 274 (4) | 1 |  |
| 1 | 122 (8) | 1.16 | 0.91 to 1.48 | 72 (5) | 1.29 | 0.95 to 1.75 |
| 2 | 43 (7) | 1.05 | 0.73 to 1.50 | 30 (5) | 1.28 | 0.83 to 1.97 |
| P-value for trend |  | 0.406 |  |  | 0.088 |  |
|  |  |  |  |  |  |  |
| *Risk structure* |  |  |  |  |  |  |
| 0 | 386 (7) | 1 |  | 231 (4) | 1 |  |
| 1 | 138 (7) | 0.92 | 0.73 to 1.16 | 87 (4) | 0.96 | 0.72 to 1.29 |
| 2 | 103 (7) | 1.02 | 0.79 to 1.33 | 58 (4) | 0.97 | 0.69 to 1.37 |
| P-value for trend |  | 0.937 |  |  | 0.827 |  |
|  |  |  |  |  |  |  |
| *Smell of mold* |  |  |  |  |  |  |
| 0 | 562 (7) | 1 |  | 335 (4) | 1 |  |
| 1 | 65 (8) | 1.05 | 0.78 to 1.43 | 41 (5) | 1.14 | 0.77 to 1.67 |
| P-value |  | 0.741 |  |  | 0.515 |  |
|  |  |  |  |  |  |  |
| *Other strong smell* |  |  |  |  |  |  |
| 0 | 512 (7) | 1 |  | 297 (4) | 1 |  |
| 1 | 115 (7) | 0.95 | 0.75 to 1.21 | 79 (5) | 1.11 | 0.83 to 1.49 |
| P-value |  | 0.683 |  |  | 0.476 |  |

*Note*. OR = Odds ratio, CI = Confidence intervals

ᵃ Symptoms reported without attribution to school environment (in general)

ᵇ Symptoms reported in relation to school environment

* Number (percentage) of pupils experiencing symptoms.

IEQ problems were analyzed in separate models. All analyses were adjusted for pupils’ age, sex, asthma, hay fever, atopic rash, parental smoking, and attending Swedish-speaking school.

**Table S8. Adjusted odds ratios for the associations between IEQ indicators and skin symptoms reported by primary school pupils (n = 8775 pupils, 99 school buildings)**

|  | Skin symptoms | | | | | |
| --- | --- | --- | --- | --- | --- | --- |
|  | In generalᵃ | | | In relation to schoolᵇ | | |
| IEQ problems | n (%)* | OR | 95% CI | n (%)* | OR | 95% CI |
| *Moisture and mold damage* |  |  |  |  |  |  |
| 0 | 464 (8) | 1 |  | 208 (4) | 1 |  |
| 1 | 168 (9) | 1.05 | 0.87 to 1.28 | 86 (4) | 1.20 | 0.90 to 1.60 |
| 2 | 111 (10) | **1.28** | **1.01 to 1.61** | 45 (4) | 1.03 | 0.72 to 1.51 |
| P-value for trend |  | 0.055 |  |  | 0.521 |  |
|  |  |  |  |  |  |  |
| *Ventilation* |  |  |  |  |  |  |
| 0 | 410 (8) | 1 |  | 181 (4) | 1 |  |
| 1 | 183 (9) | 1.03 | 0.85 to 1.25 | 82 (4) | 1.01 | 0.76 to 1.35 |
| 2 | 150 (10) | **1.27** | **1.03 to 1.57** | 76 (5) | **1.39** | **1.03 to 1.87** |
| P-value for trend |  | 0.039 |  |  | 0.057 |  |
|  |  |  |  |  |  |  |
| *Temperature* |  |  |  |  |  |  |
| 0 | 556 (8) | 1 |  | 249 (4) | 1 |  |
| 1 | 138 (10) | **1.26** | **1.03 to 1.55** | 68 (5) | **1.38** | **1.02 to 1.86** |
| 2 | 49 (8) | 0.99 | 0.72 to 1.37 | 22 (4) | 0.95 | 0.59 to 1.53 |
| P-value for trend |  | 0.281 |  |  | 0.382 |  |
|  |  |  |  |  |  |  |
| *Risk structure* |  |  |  |  |  |  |
| 0 | 451 (9) | 1 |  | 206 (4) | 1 |  |
| 1 | 170 (8) | 0.97 | 0.79 to 1.20 | 78 (4) | 0.93 | 0.69 to 1.26 |
| 2 | 122 (9) | 0.98 | 0.78 to 1.24 | 55 (4) | 0.92 | 0.65 to 1.30 |
| P-value for trend |  | 0.828 |  |  | 0.576 |  |
|  |  |  |  |  |  |  |
| *Smell of mold* |  |  |  |  |  |  |
| 0 | 652 (8) | 1 |  | 300 (4) | 1 |  |
| 1 | 91 (11) | **1.42** | **1.11 to 1.82** | 39 (5) | 1.28 | 0.87 to 1.88 |
| P-value |  | **0.005** |  |  | 0.205 |  |
|  |  |  |  |  |  |  |
| *Other strong smell* |  |  |  |  |  |  |
| 0 | 584 (8) | 1 |  | 261 (4) | 1 |  |
| 1 | 159 (9) | 1.19 | 0.98 to 1.46 | 78 (%) | 1.24 | 0.93 to 1.66 |
| P-value |  | 0.082 |  |  | 0.147 |  |

*Note*. OR = Odds ratio, CI = Confidence intervals

ᵃ Symptoms reported without attribution to school environment (in general)

ᵇ Symptoms reported in relation to school environment

* Number (percentage) of pupils experiencing symptoms.

IEQ problems were analyzed in separate models. All analyses were adjusted for pupils’ age, sex, asthma, hay fever, atopic rash, parental smoking, and attending Swedish-speaking school.

**Table S9. Adjusted odds ratios for the associations between IEQ indicators and respiratory symptoms reported by secondary school pupils (n = 3410 pupils, 30 school buildings)**

|  | Respiratory symptoms | | | | | |
| --- | --- | --- | --- | --- | --- | --- |
|  | In generalᵃ | | | In relation to schoolᵇ | | |
| IEQ problems | n (%)* | OR | 95% CI | n (%)* | OR | 95% CI |
| *Moisture and mold damage* |  |  |  |  |  |  |
| 0 | 315 (16) | 1 |  | 116 (6) | 1 |  |
| 1 | 148 (16) | 1.05 | 0.84 to 1.32 | 62 (7) | 1.18 | 0.76 to 1.84 |
| 2 | 120 (26) | **1.85** | **1.45 to 2.37** | 67 (15) | **2.85** | **1.71 to 4.76** |
| P-value for trend |  | **0.001** |  |  | **0.001** |  |
|  |  |  |  |  |  |  |
| *Ventilation* |  |  |  |  |  |  |
| 0 | 313 (16) | 1 |  | 128 (7) | 1 |  |
| 1 | 128 (15) | 0.94 | 0.69 to 1.27 | 43 (5) | 0.71 | 0.40 to 1.28 |
| 2 | 142 (22) | 1.28 | 0.93 to 1.75 | 74 (12) | 1.45 | 0.83 to 2.53 |
| P-value for trend |  | 0.254 |  |  | 0.448 |  |
|  |  |  |  |  |  |  |
| *Temperature* |  |  |  |  |  |  |
| 0 | 381 (16) | 1 |  | 146 (6) | 1 |  |
| 1 | 122 (16) | 0.99 | 0.73 to 1.34 | 57 (7) | 1.21 | 0.69 to 2.09 |
| 2 | 80 (24) | **1.59** | **1.05 to 2.39** | 42 (13) | 2.17 | 0.97 to 4.83 |
| P-value for trend |  | 0.092 |  |  | 0.066 |  |
|  |  |  |  |  |  |  |
| *Risk structure* |  |  |  |  |  |  |
| 0 | 286 (16) | 1 |  | 110 (6) | 1 |  |
| 1 | 102 (15) | 0.95 | 0.70 to 1.29 | 37 (5) | 0.84 | 0.47 to 1.49 |
| 2 | 195 (21) | **1.45** | **1.11 to 1.89** | 98 (11) | **1.90** | **1.14 to 3.17** |
| P-value for trend |  | **0.015** |  |  | **0.038** |  |
|  |  |  |  |  |  |  |
| *Smell of mold* |  |  |  |  |  |  |
| 0 | 473 (17) | 1 |  | 179 (6) | 1 |  |
| 1 | 110 (18) | 1.13 | 0.81 to 1.57 | 66 (11) | **1.84** | **1.08 to 3.15** |
| P-value |  | 0.484 |  |  | **0.025** |  |
|  |  |  |  |  |  |  |
| *Other strong smell* |  |  |  |  |  |  |
| 0 | 489 (17) | 1 |  | 193 (7) | 1 |  |
| 1 | 94 (19) | 1.05 | 0.74 to 1.50 | 52 (11) | 1.35 | 0.74 to 2.47 |
| P-value |  | 0.765 |  |  | 0.332 |  |

*Note*. OR = Odds ratio, CI = Confidence intervals

ᵃ Symptoms reported without attribution to school environment (in general)

ᵇ Symptoms reported in relation to school environment

* Number (percentage) of pupils experiencing symptoms.

IEQ problems were analyzed in separate models. All analyses were adjusted for pupils’ age, sex, asthma, hay fever, atopic rash, parental smoking, and attending Swedish-speaking school.

**Table S10. Adjusted odds ratios for the associations between IEQ indicators and general symptoms reported by secondary school pupils (n = 3410 pupils, 30 school buildings)**

|  | General symptoms | | | | | |
| --- | --- | --- | --- | --- | --- | --- |
|  | In generalᵃ | | | In relation to schoolᵇ | | |
| IEQ problems | n (%)* | OR | 95% CI | n (%)* | OR | 95% CI |
| *Moisture and mold damage* |  |  |  |  |  |  |
| 0 | 1174 (58) | 1 |  | 590 (29) | 1 |  |
| 1 | 493 (53) | 0.85 | 0.72 to 1.00 | 236 (25) | 0.88 | 0.72 to 1.08 |
| 2 | 307 (68) | **1.49** | **1.20 to 1.87** | 186 (41) | **1.71** | **1.33 to 2.19** |
| P-value for trend |  | 0.165 |  |  | **0.010** |  |
|  |  |  |  |  |  |  |
| *Ventilation* |  |  |  |  |  |  |
| 0 | 1105 (57) | 1 |  | 570 (30) | 1 |  |
| 1 | 460 (55) | 0.92 | 0.74 to 1.15 | 208 (25) | 0.83 | 0.65 to 1.06 |
| 2 | 409 (64) | 1.22 | 0.97 to 1.55 | 234 (36) | 1.33 | 1.04 to 1.69 |
| P-value for trend |  | 0.238 |  |  | 0.185 |  |
|  |  |  |  |  |  |  |
| *Temperature* |  |  |  |  |  |  |
| 0 | 1344 (58) | 1 |  | 671 (29) | 1 |  |
| 1 | 417 (55) | 0.90 | 0.71 to 1.13 | 222 (29) | 1.09 | 0.82 to 1.44 |
| 2 | 213 (65) | 1.25 | 0.89 to 1.75 | 119 (36) | 1.33 | 0.88 to 2.00 |
| P-value for trend |  | 0.624 |  |  | 0.165 |  |
|  |  |  |  |  |  |  |
| *Risk structure* |  |  |  |  |  |  |
| 0 | 1049 (58) | 1 |  | 524 (29) | 1 |  |
| 1 | 538 (52) | 0.81 | 0.66 to 0.99 | 170 (25) | 0.87 | 0.68 to 1.12 |
| 2 | 567 (62) | **1.25** | **1.04 to 1.50** | 318 (35) | **1.45** | **1.15 to 1.82** |
| P-value for trend |  | 0.141 |  |  | **0.009** |  |
|  |  |  |  |  |  |  |
| *Smell of mold* |  |  |  |  |  |  |
| 0 | 1634 (58) | 1 |  | 808 (29) | 1 |  |
| 1 | 340 (57) | 0.93 | 0.73 to 1.19 | 204 (34) | 1.32 | 1.00 to 1.75 |
| P-value |  | 0.568 |  |  | 0.052 |  |
|  |  |  |  |  |  |  |
| *Other strong smell* |  |  |  |  |  |  |
| 0 | 1659 (57) | 1 |  | 853 (29) | 1 |  |
| 1 | 315 (64) | 1.25 | 0.96 to 1.62 | 159 (32) | 1.04 | 0.76 to 1.41 |
| P-value |  | 0.100 |  |  | 0.824 |  |

*Note*. OR = Odds ratio, CI = Confidence intervals

ᵃ Symptoms reported without attribution to school environment (in general)

ᵇ Symptoms reported in relation to school environment

* Number (percentage) of pupils experiencing symptoms.

IEQ problems were analyzed in separate models. All analyses were adjusted for pupils’ age, sex, asthma, hay fever, atopic rash, parental smoking, and attending Swedish-speaking school.

**Table S11. Adjusted odds ratios for the associations between IEQ indicators and lower respiratory symptoms reported by secondary school pupils (n = 3410 pupils, 30 school buildings)**

|  | Lower respiratory symptoms | | | | | |
| --- | --- | --- | --- | --- | --- | --- |
|  | In generalᵃ | | | In relation to schoolᵇ | | |
| IEQ problems | n (%)* | OR | 95% CI | n (%)* | OR | 95% CI |
| *Moisture and mold damage* |  |  |  |  |  |  |
| 0 | 179 (9) | 1 |  | 37 (2) | 1 |  |
| 1 | 83 (9) | 0.96 | 0.72 to 1.29 | 18 (2) | 0.90 | 0.40 to 2.04 |
| 2 | 58 (13) | **1.42** | **1.02 to 1.97** | 22 (5) | **2.86** | **1.13 to 7.26** |
| P-value for trend |  | 0.099 |  |  | 0.083 |  |
|  |  |  |  |  |  |  |
| *Ventilation* |  |  |  |  |  |  |
| 0 | 175 (9) | 1 |  | 42 (2) | 1 |  |
| 1 | 74 (9) | 0.94 | 0.69 to 1.28 | 12 (1) | 0.58 | 0.22 to 1.48 |
| 2 | 71 (11) | 1.13 | 0.82 to 1.54 | 23 (4) | 1.40 | 0.59 to 3.32 |
| P-value for trend |  | 0.577 |  |  | 0.728 |  |
|  |  |  |  |  |  |  |
| *Temperature* |  |  |  |  |  |  |
| 0 | 206 (9) | 1 |  | 48 (2) | 1 |  |
| 1 | 70 (9) | 1.00 | 0.74 to 1.35 | 13 (2) | 0.65 | 0.26 to 1.60 |
| 2 | 44 (13) | **1.51** | **1.05 to 2.19** | 16 (5) | 2.80 | 0.88 to 8.93 |
| P-value for trend |  | 0.072 |  |  | 0.380 |  |
|  |  |  |  |  |  |  |
| *Risk structure* |  |  |  |  |  |  |
| 0 | 162 (9) | 1 |  | 35 (2) | 1 |  |
| 1 | 55 (8) | 0.84 | 0.60 to 1.18 | 8 (1) | 0.50 | 0.19 to 1.36 |
| 2 | 103 (11) | 1.25 | 0.95 to 1.65 | 34 (4) | 1.98 | 0.92 to 4.28 |
| P-value for trend |  | 0.151 |  |  | 0.188 |  |
|  |  |  |  |  |  |  |
| *Smell of mold* |  |  |  |  |  |  |
| 0 | 264 (9) | 1 |  | 57 (2) | 1 |  |
| 1 | 56 (9) | 0.96 | 0.69 to 1.33 | 20 (3) | 1.52 | 0.62 to 3.72 |
| P-value |  | 0.789 |  |  | 0.362 |  |
|  |  |  |  |  |  |  |
| *Other strong smell* |  |  |  |  |  |  |
| 0 | 262 (9) | 1 |  | 61 (2) | 1 |  |
| 1 | 58 (12) | 1.25 | 0.91 to 1.72 | 16 (3) | 1.27 | 0.49 to 3.34 |
| P-value |  | 0.163 |  |  | 0.622 |  |

*Note*. OR = Odds ratio, CI = Confidence intervals

ᵃ Symptoms reported without attribution to school environment (in general)

ᵇ Symptoms reported in relation to school environment

* Number (percentage) of pupils experiencing symptoms.

IEQ problems were analyzed in separate models. All analyses were adjusted for pupils’ age, sex, asthma, hay fever, atopic rash, parental smoking, and attending Swedish-speaking school.

**Table S12. Adjusted odds ratios for the associations between IEQ indicators and eye symptoms reported by secondary school pupils (n = 3410 pupils, 30 school buildings)**

|  | Eye symptoms | | | | | |
| --- | --- | --- | --- | --- | --- | --- |
|  | In generalᵃ | | | In relation to schoolᵇ | | |
| IEQ problems | n (%)* | OR | 95% CI | n (%)* | OR | 95% CI |
| *Moisture and mold damage* |  |  |  |  |  |  |
| 0 | 342 (17) | 1 |  | 166 (8) | 1 |  |
| 1 | 134 (14) | 0.80 | 0.64 to 1.00 | 61 (7) | 0.77 | 0.53 to 1.12 |
| 2 | 93 (20) | 1.24 | 0.95 to 1.61 | 53 (12) | 1.52 | 1.00 to 2.30 |
| P-value for trend |  | 0.562 |  |  | 0.293 |  |
|  |  |  |  |  |  |  |
| *Ventilation* |  |  |  |  |  |  |
| 0 | 318 (17) | 1 |  | 160 (8) | 1 |  |
| 1 | 136 (16) | 0.94 | 0.75 to 1.19 | 60 (7) | 0.85 | 0.57 to 1.28 |
| 2 | 115 (18) | 1.04 | 0.82 to 1.32 | 60 (9) | 1.06 | 0.71 to 1.60 |
| P-value for trend |  | 0.860 |  |  | 0.974 |  |
|  |  |  |  |  |  |  |
| *Temperature* |  |  |  |  |  |  |
| 0 | 394 (17) | 1 |  | 184 (8) | 1 |  |
| 1 | 111 (15) | 0.80 | 0.63 to 1.02 | 57 (7) | 0.95 | 0.65 to 1.40 |
| 2 | 64 (19) | 1.17 | 0.86 to 1.58 | 39 (12) | 1.56 | 0.95 to 2.57 |
| P-value for trend |  | 0.941 |  |  | 0.207 |  |
|  |  |  |  |  |  |  |
| *Risk structure* |  |  |  |  |  |  |
| 0 | 311 (17) | 1 |  | 150 (8) | 1 |  |
| 1 | 94 (14) | 0.73 | 0.56 to 0.94 | 42 (6) | 0.73 | 0.47 to 1.11 |
| 2 | 164 (18) | 1.03 | 0.83 to 1.28 | 88 (10) | 1.24 | 0.87 to 1.77 |
| P-value for trend |  | 0.990 |  |  | 0.401 |  |
|  |  |  |  |  |  |  |
| *Smell of mold* |  |  |  |  |  |  |
| 0 | 468 (17) | 1 |  | 220 (8) | 1 |  |
| 1 | 101 (17) | 1.03 | 0.81 to 1.31 | 60 (10) | 1.33 | 0.91 to 1.94 |
| P-value |  | 0.798 |  |  | 0.143 |  |
|  |  |  |  |  |  |  |
| *Other strong smell* |  |  |  |  |  |  |
| 0 | 476 (16) | 1 |  | 227 (8) | 1 |  |
| 1 | 93 (19) | 1.12 | 0.87 to 1.44 | 53 (11) | 1.32 | 0.89 to 1.95 |
| P-value |  | 0.377 |  |  | 0.167 |  |

*Note*. OR = Odds ratio, CI = Confidence intervals

ᵃ Symptoms reported without attribution to school environment (in general)

ᵇ Symptoms reported in relation to school environment

* Number (percentage) of pupils experiencing symptoms.

IEQ problems were analyzed in separate models. All analyses were adjusted for pupils’ age, sex, asthma, hay fever, atopic rash, parental smoking, and attending Swedish-speaking school.

**Table S13. Adjusted odds ratios for the associations between IEQ indicators and skin symptoms reported by secondary school pupils (n = 3410 pupils, 30 school buildings)**

|  | Skin symptoms | | | | | |
| --- | --- | --- | --- | --- | --- | --- |
|  | In generalᵃ | | | In relation to schoolᵇ | | |
| IEQ problems | n (%)* | OR | 95% CI | n (%)* | OR | 95% CI |
| *Moisture and mold damage* |  |  |  |  |  |  |
| 0 | 123 (6) | 1 |  | 28 (1.3) | 1 |  |
| 1 | 47 (5) | 0.95 | 0.65 to 1.39 | 14 (1.5) | 1.26 | 0.63 to 2.51 |
| 2 | 37 (8) | 1.28 | 0.84 to 1.96 | 14 (3) | 1.98 | 0.95 to 4.14 |
| P-value for trend |  | 0.376 |  |  | 0.054 |  |
|  |  |  |  |  |  |  |
| *Ventilation* |  |  |  |  |  |  |
| 0 | 121 (6) | 1 |  | 26 (1) | 1 |  |
| 1 | 42 (5) | 0.80 | 0.54 to 1.20 | 14 (2) | 1.43 | 0.72 to 2.86 |
| 2 | 44 (7) | 1.12 | 0.76 to 1.66 | 16 (2.5) | 1.80 | 0.95 to 3.43 |
| P-value for trend |  | 0.825 |  |  | 0.066 |  |
|  |  |  |  |  |  |  |
| *Temperature* |  |  |  |  |  |  |
| 0 | 148 (6) | 1 |  | 33 (1.4) | 1 |  |
| 1 | 37 (5) | 0.79 | 0.53 to 1.18 | 14 (2) | 1.48 | 0.76 to 2.87 |
| 2 | 22 (7) | 1.02 | 0.61 to 1.70 | 9 (3) | 1.81 | 0.83 to 3.96 |
| P-value for trend |  | 0.613 |  |  | 0.081 |  |
|  |  |  |  |  |  |  |
| *Risk structure* |  |  |  |  |  |  |
| 0 | 118 (6) | 1 |  | 26 (1.4) | 1 |  |
| 1 | 30 (4) | 0.74 | 0.47 to 1.17 | 8 (1.2) | 0.94 | 0.41 to 2.16 |
| 2 | 62 (7) | 1.10 | 0.77 to 1.56 | 22 (2.4) | 1.68 | 0.93 to 3.03 |
| P-value for trend |  | 0.735 |  |  | 0.094 |  |
|  |  |  |  |  |  |  |
| *Smell of mold* |  |  |  |  |  |  |
| 0 | 174 (6) | 1 |  | 41 (1.5) | 1 |  |
| 1 | 33 (6) | 1.05 | 0.69 to 1.60 | 15 (2.5) | **1.98** | **1.07 to 3.68** |
| P-value |  | 0.821 |  |  | **0.030** |  |
|  |  |  |  |  |  |  |
| *Other strong smell* |  |  |  |  |  |  |
| 0 | 173 (6) | 1 |  | 42 (1.4) | 1 |  |
| 1 | 34 (7) | 1.03 | 0.68 to 1.57 | 14 (3) | 1.86 | 0.99 to 3.50 |
| P-value |  | 0.882 |  |  | 0.055 |  |

*Note*. OR = Odds ratio, CI = Confidence intervals

ᵃ Symptoms reported without attribution to school environment (in general)

ᵇ Symptoms reported in relation to school environment

* Number (percentage) of pupils experiencing symptoms.

IEQ problems were analyzed in separate models. All analyses were adjusted for pupils’ age, sex, asthma, hay fever, atopic rash, parental smoking, and attending Swedish-speaking school.

**Table S14. Adjusted odds ratios for the associations between indoor environmental quality (IEQ) and symptoms reported by parents for their primary school pupils (n = 3540 pupils, 88 school buildings)**

|  | In generalᵃ | | |  | In relation to schoolᵇ | | |
| --- | --- | --- | --- | --- | --- | --- | --- |
| Symptom score | n (%)** | OR | 95% CI |  | n (%) | OR | 95% CI |
| *Respiratory* |  |  |  |  |  |  |  |
| Good IEQ | 158 (11) | 1.00 |  |  | 33 (2) | 1.00 |  |
| Moderate IEQ | 159 (12) | 1.13 | 0.86 to 1.49 |  | 66 (5) | **2.50** | **1.52 to 4.13** |
| Poor IEQ | 127 (17) | **1.61** | **1.18 to 2.19** |  | 63 (8) | **4.19** | **1.37 to 2.19** |
| IEQ summary score* |  | **1.07** | **1.03 to 1.11** |  |  | **1.17** | **1.09 to 1.25** |
|  |  |  |  |  |  |  |  |
| *Lower respiratory* |  |  |  |  |  |  |  |
| Good IEQ | 78 (5) | 1.00 |  |  | 20 (1) | 1.00 |  |
| Moderate IEQ | 71 (5) | 0.95 | 0.68 to 1.34 |  | 17 (1) | 0.87 | 0.42 to 1.79 |
| Poor IEQ | 65 (9) | **1.50** | **1.06 to 2.14** |  | 25 (3) | **2.30** | **1.10 to 4.78** |
| IEQ summary score* |  | **1.05** | **1.01 to 1.10** |  |  | **1.13** | **1.03 to 1.24** |
|  |  |  |  |  |  |  |  |
| *Eye* |  |  |  |  |  |  |  |
| Good IEQ | 95 (6) | 1.00 |  |  | 39 (3) | 1.00 |  |
| Moderate IEQ | 100 (8) | 1.18 | 0.85 to 1.64 |  | 50 (4) | 1.47 | 0.89 to 2.41 |
| Poor IEQ | 68 (9) | 1.37 | 0.94 to 2.00 |  | 42 (6) | **2.03** | **1.17 to 3.53** |
| IEQ summary score* |  | 1.02 | 0.98 to 1.08 |  |  | 1.06 | 0.98 to 1.14 |
|  |  |  |  |  |  |  |  |
| *Skin* |  |  |  |  |  |  |  |
| Good IEQ | 114 (8) | 1.00 |  |  | 18 (1) | 1.00 |  |
| Moderate IEQ | 115 (9) | 1.23 | 0.91 to 1.67 |  | 21 (2) | 1.43 | 0.68 to 2.98 |
| Poor IEQ | 80 (11) | **1.54** | **1.10 to 2.16** |  | 13 (2) | 1.55 | 0.65 to 3.68 |
| IEQ summary score* |  | 1.04 | 1.00 to 1.09 |  |  | 1.03 | 0.982to 1.15 |
|  |  |  |  |  |  |  |  |
| *General* |  |  |  |  |  |  |  |
| Good IEQ | 350 (24) | 1.00 |  |  | 112 (8) | 1.00 |  |
| Moderate IEQ | 373 (28) | **1.28** | **1.04 to 1.57** |  | 164 (13) | **1.78** | **1.31 to 2.44** |
| Poor IEQ | 232 (31) | **1.42** | **1.11 to 1.81** |  | 121 (16) | **2.38** | **1.66 to 3.42** |
| IEQ summary score* |  | **1.04** | **1.01 to 1.07** |  |  | **1.10** | **1.05 to 1.15** |

*Note*. OR = Odds ratio, CI = Confidence intervals

ᵃ Symptoms reported without attribution to school environment (in general)

ᵇ Symptoms reported in relation to school environment

* IEQ summary score (0-10) is used as a continuous variable

** Number (percentage) of pupils experiencing symptoms is reported per each latent class of IEQ. The total number of pupils in each latent class is the following: Good IEQ: n = 1468 (42%), Moderate IEQ: n = 1312 (37%), Poor IEQ: n = 760 (21%)

All analyses were adjusted for pupils’ age, sex, asthma, hay fever, atopic rash, parental smoking, and attending Swedish-speaking school.

Separate models were tested for each symptom score.

**Table S15. Adjusted odds ratios for the associations between IEQ indicators and respiratory symptoms reported by parents for their primary school pupils (n = 3540 parents, 88 school buildings)**

|  | Respiratory symptoms | | | | | |
| --- | --- | --- | --- | --- | --- | --- |
|  | In generalᵃ | | | In relation to schoolᵇ | | |
| IEQ problems | n (%)* | OR | 95% CI | n (%)* | OR | 95% CI |
| *Moisture and mold damage* |  |  |  |  |  |  |
| 0 | 249 (12) | 1 |  | 70 (3) | 1 |  |
| 1 | 113 (12) | 1.02 | 0.76 to 1.36 | 54 (6) | **1.82** | **1.10 to 3.01** |
| 2 | 82 (18) | **1.55** | **1.10 to 2.20** | 38 (8) | **2.79** | **1.52 to 5.12** |
| P-value for trend |  | **0.038** |  |  | **0.001** |  |
|  |  |  |  |  |  |  |
| *Ventilation* |  |  |  |  |  |  |
| 0 | 209 (11) | 1 |  | 52 (3) | 1 |  |
| 1 | 105 (13) | 1.15 | 0.86 to 1.55 | 45 (5) | **2.20** | **1.34 to 3.59** |
| 2 | 130 (16) | **1.52** | **1.13 to 2.04** | 65 (8) | **3.31** | **2.03 to 5.38** |
| P-value for trend |  | **0.006** |  |  | **0.001** |  |
|  |  |  |  |  |  |  |
| *Temperature* |  |  |  |  |  |  |
| 0 | 282 (11) | 1 |  | 79 (3) | 1 |  |
| 1 | 96 (16) | **1.51** | **1.12 to 2.04** | 55 (9) | **3.12** | **2.03 to 4.81** |
| 2 | 66 (17) | **1.75** | **1.21 to 2.52** | 28 (7) | **2.71** | **1.54 to 4.77** |
| P-value for trend |  | **0.001** |  |  | **0.001** |  |
|  |  |  |  |  |  |  |
| *Risk structure* |  |  |  |  |  |  |
| 0 | 238 (11) | 1 |  | 75 (4) | 1 |  |
| 1 | 104 (13) | 1.11 | 0.83 to 1.50 | 38 (5) | 1.35 | 0.79 to 2.31 |
| 2 | 102 (17) | **1.55** | **1.13 to 2.12** | 49 (8) | **2.52** | **1.44 to 4.39** |
| P-value for trend |  | **0.010** |  |  | **0.002** |  |
|  |  |  |  |  |  |  |
| *Smell of mold* |  |  |  |  |  |  |
| 0 | 375 (12) | 1 |  | 134 (4) | 1 |  |
| 1 | 69 (19) | **1.63** | **1.14 to 2.33** | 28 (8) | 1.71 | 0.85 to 3.44 |
| P-value |  | **0.008** |  |  | 0.130 |  |
|  |  |  |  |  |  |  |
| *Other strong smell* |  |  |  |  |  |  |
| 0 | 315 (12) | 1 |  | 104 (4) | 1 |  |
| 1 | 129 (15) | 1.22 | 0.91 to 1.62 | 58 (7) | 1.67 | 1.00 to 2.78 |
| P-value |  | 0.183 |  |  | 0.048 |  |

*Note*. OR = Odds ratio, CI = Confidence intervals

ᵃ Symptoms reported without attribution to school environment (in general)

ᵇ Symptoms reported in relation to school environment

* Number (percentage) of pupils experiencing symptoms.

IEQ problems were analyzed in separate models. All analyses were adjusted for pupils’ age, sex, asthma, hay fever, atopic rash, parental smoking, and attending Swedish-speaking school.

**Table S16. Adjusted odds ratios for the associations between IEQ indicators and general symptoms reported by parents for their primary school pupils (n = 3540 parents, 88 school buildings)**

|  | General symptoms | | | | | |
| --- | --- | --- | --- | --- | --- | --- |
|  | In generalᵃ | | | In relation to schoolᵇ | | |
| IEQ problems | n (%)* | OR | 95% CI | n (%)* | OR | 95% CI |
| *Moisture and mold damage* |  |  |  |  |  |  |
| 0 | 549 (26) | 1 |  | 193 (9) | 1 |  |
| 1 | 268 (28) | 1.16 | 0.93 to 1.45 | 140 (15) | **1.87** | **1.36 to 2.58** |
| 2 | 138 (30) | 1.25 | 0.94 to 1.66 | 64 (14) | **1.68** | **1.10 to 2.56** |
| P-value for trend |  | 0.070 |  |  | **0.001** |  |
|  |  |  |  |  |  |  |
| *Ventilation* |  |  |  |  |  |  |
| 0 | 475 (25) | 1 |  | 173 (9) | 1 |  |
| 1 | 248 (30) | **1.29** | **1.03 to 1.61** | 106 (13) | **1.51** | **1.07 to 2.13** |
| 2 | 232 (29) | 1.21 | 0.96 to 1.53 | 118 (15) | **1.69** | **1.18 to 2.43** |
| P-value for trend |  | 0.050 |  |  | **0.002** |  |
|  |  |  |  |  |  |  |
| *Temperature* |  |  |  |  |  |  |
| 0 | 657 (26) | 1 |  | 240 (9) | 1 |  |
| 1 | 187 (32) | **1.31** | **1.03 to 1.67** | 103 (18) | **1.87** | **1.32 to 2.64** |
| 2 | 111 (29) | 1.29 | 0.94 to 1.76 | 54 (14) | **1.87** | **1.19 to 2.92** |
| P-value for trend |  | **0.024** |  |  | **0.001** |  |
|  |  |  |  |  |  |  |
| *Risk structure* |  |  |  |  |  |  |
| 0 | 551 (26) | 1 |  | 207 (10) | 1 |  |
| 1 | 221 (28) | 1.03 | 0.81 to 1.30 | 94 (12) | 1.15 | 0.80 to 1.64 |
| 2 | 183 (31) | 1.27 | 0.98 to 1.64 | 96 (16) | **1.87** | **1.27 to 2.75** |
| P-value for trend |  | 0.098 |  |  | **0.002** |  |
|  |  |  |  |  |  |  |
| *Smell of mold* |  |  |  |  |  |  |
| 0 | 831 (26) | 1 |  | 334 (11) | 1 |  |
| 1 | 124 (34) | **1.44** | **1.08 to 1.94** | 63 (17) | **1.75** | **1.11 to 2.76** |
| P-value |  | **0.015** |  |  | **0.017** |  |
|  |  |  |  |  |  |  |
| *Other strong smell* |  |  |  |  |  |  |
| 0 | 703 (26) | 1 |  | 286 (11) | 1 |  |
| 1 | 252 (29) | 1.12 | 0.90 to 1.40 | 111 (13) | 1.08 | 0.75 to 1.56 |
| P-value |  | 0.308 |  |  | 0.665 |  |

*Note*. OR = Odds ratio, CI = Confidence intervals

ᵃ Symptoms reported without attribution to school environment (in general)

ᵇ Symptoms reported in relation to school environment

* Number (percentage) of pupils experiencing symptoms.

IEQ problems were analyzed in separate models. All analyses were adjusted for pupils’ age, sex, asthma, hay fever, atopic rash, parental smoking, and attending Swedish-speaking school.

**Table S17. Adjusted odds ratios for the associations between IEQ indicators and lower respiratory symptoms reported by parents for their primary school pupils (n = 3540 parents, 88 school buildings)**

|  | Lower respiratory symptoms | | | | | |
| --- | --- | --- | --- | --- | --- | --- |
|  | In generalᵃ | | | In relation to schoolᵇ | | |
| IEQ problems | n (%)* | OR | 95% CI | n (%)* | OR | 95% CI |
| *Moisture and mold damage* |  |  |  |  |  |  |
| 0 | 118 (6) | 1 |  | 26 (1) | 1 |  |
| 1 | 58 (6) | 1.08 | 0.76 to 1.54 | 21 (2) | 1.84 | 0.94 to 3.59 |
| 2 | 38 (8) | 1.37 | 0.91 to 2.08 | 15 (3) | **2.40** | **1.10 to 5.22** |
| P-value for trend |  | 0.158 |  |  | **0.016** |  |
|  |  |  |  |  |  |  |
| *Ventilation* |  |  |  |  |  |  |
| 0 | 103 (5) | 1 |  | 25 (1) | 1 |  |
| 1 | 49 (6) | 1.07 | 0.74 to 1.54 | 13 (2) | 1.25 | 0.58 to 2.69 |
| 2 | 62 (8) | 1.38 | 0.98 to 1.93 | 24 (3) | **2.31** | **1.15 to 4.62** |
| P-value for trend |  | 0.078 |  |  | **0.021** |  |
|  |  |  |  |  |  |  |
| *Temperature* |  |  |  |  |  |  |
| 0 | 135 (5) | 1 |  | 32 (1) | 1 |  |
| 1 | 46 (8) | **1.48** | **1.04 to 2.12** | 18 (3) | **2.37** | **1.21 to 4.66** |
| 2 | 33 (9) | **1.64** | **1.09 to 2.48** | 12 (3) | **2.77** | **1.20 to 6.39** |
| P-value for trend |  | **0.006** |  |  | **0.004** |  |
|  |  |  |  |  |  |  |
| *Risk structure* |  |  |  |  |  |  |
| 0 | 109 (5) | 1 |  | 28 (1) | 1 |  |
| 1 | 56 (7) | 1.29 | 0.91 to 1.84 | 17 (2) | 1.56 | 0.75 to 3.26 |
| 2 | 49 (8) | **1.51** | **1.04 to 2.18** | 17 (3) | 1.93 | 0.90 to 4.13 |
| P-value for trend |  | **0.022** |  |  | 0.078 |  |
|  |  |  |  |  |  |  |
| *Smell of mold* |  |  |  |  |  |  |
| 0 | 185 (6) | 1 |  | 51 (2) | 1 |  |
| 1 | 29 (8) | 1.34 | 0.86 to 2.08 | 11 (3) | 1.82 | 0.81 to 4.08 |
| P-value |  | 0.191 |  |  | 0.146 |  |
|  |  |  |  |  |  |  |
| *Other strong smell* |  |  |  |  |  |  |
| 0 | 153 (6) | 1 |  | 40 (2) | 1 |  |
| 1 | 61 (7) | 1.15 | 0.82 to 1.61 | 22 (3) | 1.62 | 0.86 to 3.07 |
| P-value |  | 0.412 |  |  | 0.137 |  |

*Note*. OR = Odds ratio, CI = Confidence intervals

ᵃ Symptoms reported without attribution to school environment (in general)

ᵇ Symptoms reported in relation to school environment

* Number (percentage) of pupils experiencing symptoms.

IEQ problems were analyzed in separate models. All analyses were adjusted for pupils’ age, sex, asthma, hay fever, atopic rash, parental smoking, and attending Swedish-speaking school.

**Table S18. Adjusted odds ratios for the associations between IEQ indicators and eye symptoms reported by parents for their primary school pupils (n = 3540 parents, 88 school buildings)**

|  | Eye symptoms | | | | | |
| --- | --- | --- | --- | --- | --- | --- |
|  | In generalᵃ | | | In relation to schoolᵇ | | |
| IEQ problems | n (%)* | OR | 95% CI | n (%)* | OR | 95% CI |
| *Moisture and mold damage* |  |  |  |  |  |  |
| 0 | 146 (7) | 1 |  | 66 (3) | 1 |  |
| 1 | 85 (9) | 1.33 | 0.96 to 1.83 | 49 (5) | **1.72** | **1.06 to 2.79** |
| 2 | 32 (7) | 0.98 | 0.62 to 1.53 | 16 (3) | 1.03 | 0.51 to 2.06 |
| P-value for trend |  | 0.541 |  |  | 0.330 |  |
|  |  |  |  |  |  |  |
| *Ventilation* |  |  |  |  |  |  |
| 0 | 127 (7) | 1 |  | 50 (3) | 1 |  |
| 1 | 67 (8) | 1.26 | 0.89 to 1.79 | 39 (5) | **1.92** | **1.16 to 3.18** |
| 2 | 69 (9) | 1.29 | 0.90 to 1.85 | 42 (5) | **1.96** | **1.17 to 3.28** |
| P-value for trend |  | 0.120 |  |  | **0.005** |  |
|  |  |  |  |  |  |  |
| *Temperature* |  |  |  |  |  |  |
| 0 | 172 (7) | 1 |  | 70 (3) | 1 |  |
| 1 | 66 (11) | **1.75** | **1.29 to 2.38** | 42 (7) | **2.33** | **1.50 to 3.60** |
| 2 | 25 (7) | 0.98 | 0.63 to 1.52 | 10 (3) | 0.86 | 0.42 to 1.74 |
| P-value for trend |  | 0.182 |  |  | 0.247 |  |
|  |  |  |  |  |  |  |
| *Risk structure* |  |  |  |  |  |  |
| 0 | 154 (7) | 1 |  | 72 (3) | 1 |  |
| 1 | 59 (7) | 0.98 | 0.68 to 1.40 | 37 (5) | 1.30 | 0.77 to 2.20 |
| 2 | 50 (8) | 1.10 | 0.74 to 1.64 | 22 (4) | 0.97 | 0.51 to 1.85 |
| P-value for trend |  | 0.711 |  |  | 0.804 |  |
|  |  |  |  |  |  |  |
| *Smell of mold* |  |  |  |  |  |  |
| 0 | 236 (7) | 1 |  | 114 (4) | 1 |  |
| 1 | 27 (7) | 0.91 | 0.56 to 1.49 | 17 (5) | 1.15 | 0.56 to 2.33 |
| P-value |  | 0.708 |  |  | 0.706 |  |
|  |  |  |  |  |  |  |
| *Other strong smell* |  |  |  |  |  |  |
| 0 | 196 (7) | 1 |  | 91 (3) | 1 |  |
| 1 | 67 (8) | 1.01 | 0.72 to 1.42 | 40 (5) | 1.22 | 0.73 to 2.03 |
| P-value |  | 0.958 |  |  | 0.449 |  |

*Note*. OR = Odds ratio, CI = Confidence intervals

ᵃ Symptoms reported without attribution to school environment (in general)

ᵇ Symptoms reported in relation to school environment

* Number (percentage) of pupils experiencing symptoms.

IEQ problems were analyzed in separate models. All analyses were adjusted for pupils’ age, sex, asthma, hay fever, atopic rash, parental smoking, and attending Swedish-speaking school.

**Table S19. Adjusted odds ratios for the associations between IEQ indicators and skin symptoms reported by parents for their primary school pupils (n = 3540 parents, 88 school buildings)**

|  | Skin symptoms | | | | | |
| --- | --- | --- | --- | --- | --- | --- |
|  | In generalᵃ | | | In relation to schoolᵇ | | |
| IEQ problems | n (%)* | OR | 95% CI | n (%)* | OR | 95% CI |
| *Moisture and mold damage* |  |  |  |  |  |  |
| 0 | 174 (8) | 1 |  | 30 (1) | 1 |  |
| 1 | 88 (9) | 1.19 | 0.88 to 1.61 | 14 (1.5) | 1.13 | 0.52 to 2.43 |
| 2 | 47 (10) | 1.39 | 0.94 to 2.04 | 8 (2) | 1.46 | 0.56 to 3.81 |
| P-value for trend |  | 0.069 |  |  | 0.450 |  |
|  |  |  |  |  |  |  |
| *Ventilation* |  |  |  |  |  |  |
| 0 | 153 (8) | 1 |  | 21 (1) | 1 |  |
| 1 | 75 (9) | 1.24 | 0.90 to 1.72 | 17 (2) | 2.07 | 1.00 to 4.31 |
| 2 | 81 (10) | **1.43** | **1.04 to 1.97** | 14 (2) | **1.73** | **1.79 to 3.80** |
| P-value for trend |  | **0.023** |  |  | 0.107 |  |
|  |  |  |  |  |  |  |
| *Temperature* |  |  |  |  |  |  |
| 0 | 207 (8) | 1 |  | 38 (1.5) | 1 |  |
| 1 | 67 (11) | **1.76** | **1.26 to 2.47** | 12 (2) | 1.46 | 0.69 to 3.09 |
| 2 | 35 (9) | 1.05 | 0.69 to 1.60 | 2 (0.5) | 0.33 | 0.07 to 1.48 |
| P-value for trend |  | 0.139 |  |  | 0.463 |  |
|  |  |  |  |  |  |  |
| *Risk structure* |  |  |  |  |  |  |
| 0 | 182 (8) | 1 |  | 33 (1.5) | 1 |  |
| 1 | 74 (9) | 1.11 | 0.81 to 1.53 | 12 (1.5) | 0.96 | 0.44 to 2.09 |
| 2 | 53 (9) | 1.01 | 0.70 to 1.45 | 7 (1.2) | 0.82 | 0.32 to 2.10 |
| P-value for trend |  | 0.805 |  |  | 0.691 |  |
|  |  |  |  |  |  |  |
| *Smell of mold* |  |  |  |  |  |  |
| 0 | 265 (8) | 1 |  | 42 (1) | 1 |  |
| 1 | 44 (12) | **1.58** | **1.07 to 2.33** | 10 (3) | 2.25 | 0.90 to 5.58 |
| P-value |  | **0.022** |  |  | 0.081 |  |
|  |  |  |  |  |  |  |
| *Other strong smell* |  |  |  |  |  |  |
| 0 | 222 (8) | 1 |  | 40 (1.5) | 1 |  |
| 1 | 87 (10) | 1.31 | 0.97 to 1.76 | 12 (1.4) | 0.91 | 0.42 to 1.96 |
| P-value |  | 0.73 |  |  | 0.814 |  |

*Note*. OR = Odds ratio, CI = Confidence intervals

ᵃ Symptoms reported without attribution to school environment (in general)

ᵇ Symptoms reported in relation to school environment

* Number (percentage) of pupils experiencing symptoms.

IEQ problems were analyzed in separate models. All analyses were adjusted for pupils’ age, sex, asthma, hay fever, atopic rash, parental smoking, and attending Swedish-speaking school.

**Table S20**. Benjamini-Hochberg test of false discovery rate

| **IEQ measure** | **Symptom** | **Sample** | **Rank** | **Raw p-value** | **Critical value at 0.10** | **Critical value at 0.05** |
| --- | --- | --- | --- | --- | --- | --- |
| Poor IEQ | Respiratory, in relation to school | Parents | 1 | <0.001 | 0.001 | 0.001 |
| Poor IEQ | General, in relation to school | Parents | 2 | <0.001 | 0.002 | 0.001 |
| Poor IEQ | Respiratory, in relation to school | Primary pupils | 3 | <0.001 | 0.003 | 0.002 |
| IEQ sumscore | Respiratory, in relation to school | Parents | 4 | <0.001 | 0.004 | 0.002 |
| IEQ sumscore | Respiratory, in relation to school | Primary pupils | 5 | <0.001 | 0.006 | 0.003 |
| IEQ sumscore | General, in relation to school | Parents | 6 | <0.001 | 0.007 | 0.003 |
| Poor IEQ | General, in general | Primary pupils | 7 | <0.001 | 0.008 | 0.004 |
| Moderate IEQ | General, in relation to school | Parents | 8 | <0.001 | 0.009 | 0.004 |
| Moderate IEQ | Respiratory, in relation to school | Parents | 9 | <0.001 | 0.010 | 0.005 |
| IEQ sumscore | Respiratory, in general | Primary pupils | 10 | <0.001 | 0.011 | 0.006 |
| IEQ sumscore | Respiratory, in general | Parents | 11 | 0.001 | 0.012 | 0.006 |
| Poor IEQ | Respiratory, in general | Primary pupils | 12 | 0.001 | 0.013 | 0.007 |
| Poor IEQ | General, in relation to school | Primary pupils | 13 | 0.001 | 0.014 | 0.007 |
| Poor IEQ | Respiratory, in general | Parents | 14 | 0.003 | 0.016 | 0.008 |
| Poor IEQ | General, in general | Parents | 15 | 0.005 | 0.017 | 0.008 |
| Moderate IEQ | Respiratory, in general | Primary pupils | 16 | **0.009** | 0.018 | **0.009** |
| IEQ sumscore | Lower respiratory, in relation to school | Parents | 17 | 0.010 | 0.019 | 0.009 |
| IEQ sumscore | Respiratory, in relation to school | Secondary pupils | 18 | 0.011 | 0.020 | 0.010 |
| Poor IEQ | Eye, in relation to school | Parents | 19 | 0.012 | 0.021 | 0.011 |
| Poor IEQ | Skin, in general | Parents | 20 | 0.013 | 0.022 | 0.011 |
| IEQ sumscore | General, in general | Primary pupils | 21 | 0.013 | 0.023 | 0.012 |
| Poor IEQ | Skin, in relation to school | Secondary pupils | 22 | 0.014 | 0.024 | 0.012 |
| IEQ sumscore | Respiratory, in general | Secondary pupils | 23 | 0.017 | 0.026 | 0.013 |
| IEQ sumscore | General, in general | Parents | 24 | 0.018 | 0.027 | 0.013 |
| Poor IEQ | Skin, in general | Primary pupils | 25 | 0.018 | 0.028 | 0.014 |
| Moderate IEQ | General, in general | Parents | 26 | 0.019 | 0.029 | 0.014 |
| IEQ sumscore | General, in relation to school | Secondary pupils | 27 | 0.019 | 0.030 | 0.015 |
| IEQ sumscore | Lower respiratory, in general | Parents | 28 | 0.020 | 0.031 | 0.016 |
| IEQ sumscore | General, in relation to school | Primary pupils | 29 | 0.021 | 0.032 | 0.016 |
| IEQ sumscore | Skin, in relation to school | Secondary pupils | 30 | 0.021 | 0.033 | 0.017 |
| Poor IEQ | Skin, in relation to school | Primary pupils | 31 | 0.023 | 0.034 | 0.017 |
| Poor IEQ | Lower respiratory, in general | Parents | 32 | 0.024 | 0.036 | 0.018 |
| Poor IEQ | Lower respiratory, in relation to school | Parents | 33 | 0.027 | 0.037 | 0.018 |
| Moderate IEQ | Respiratory, in relation to school | Primary pupils | 34 | **0.031** | **0.038** | 0.019 |
| IEQ sumscore | Skin, in general | Parents | 35 | 0.051 | 0.039 | 0.019 |
| Moderate IEQ | Skin, in relation to school | Secondary pupils | 36 | 0.052 | 0.040 | 0.020 |
| IEQ sumscore | Skin, in general | Primary pupils | 37 | 0.070 | 0.041 | 0.021 |
| Poor IEQ | Lower respiratory, in general | Primary pupils | 38 | 0.075 | 0.042 | 0.021 |
| Poor IEQ | Eye, in relation to school | Primary pupils | 39 | 0.089 | 0.043 | 0.022 |
| Poor IEQ | Eye, in general | Parents | 40 | 0.101 | 0.044 | 0.022 |
| IEQ sumscore | Lower respiratory, in general | Secondary pupils | 41 | 0.125 | 0.046 | 0.023 |
| Moderate IEQ | Eye, in relation to school | Parents | 42 | 0.132 | 0.047 | 0.023 |
| IEQ sumscore | Eye, in relation to school | Parents | 43 | 0.135 | 0.048 | 0.024 |
| IEQ sumscore | Lower respiratory, in relation to school | Primary pupils | 44 | 0.136 | 0.049 | 0.024 |
| Poor IEQ | Lower respiratory, in relation to school | Primary pupils | 45 | 0.137 | 0.050 | 0.025 |
| Poor IEQ | Respiratory, in relation to school | Secondary pupils | 46 | 0.137 | 0.051 | 0.026 |
| IEQ sumscore | Lower respiratory, in general | Primary pupils | 47 | 0.159 | 0.052 | 0.026 |
| Moderate IEQ | Skin, in general | Parents | 48 | 0.183 | 0.053 | 0.027 |
| IEQ sumscore | General, in general | Secondary pupils | 49 | 0.196 | 0.054 | 0.027 |
| IEQ sumscore | Lower respiratory, in relation to school | Secondary pupils | 50 | 0.210 | 0.056 | 0.028 |
| Poor IEQ | Respiratory, in general | Secondary pupils | 51 | 0.216 | 0.057 | 0.028 |
| Moderate IEQ | General, in relation to school | Secondary pupils | 52 | 0.235 | 0.058 | 0.029 |
| IEQ sumscore | Eye, in relation to school | Secondary pupils | 53 | 0.255 | 0.059 | 0.029 |
| IEQ sumscore | Eye, in relation to school | Primary pupils | 54 | 0.262 | 0.060 | 0.030 |
| IEQ sumscore | Skin, in relation to school | Primary pupils | 55 | 0.286 | 0.061 | 0.031 |
| Poor IEQ | Lower respiratory, in general | Secondary pupils | 56 | 0.291 | 0.062 | 0.031 |
| Poor IEQ | Skin, in relation to school | Parents | 57 | 0.323 | 0.063 | 0.032 |
| Moderate IEQ | Eye, in general | Parents | 58 | 0.325 | 0.064 | 0.032 |
| Moderate IEQ | Eye, in relation to school | Secondary pupils | 59 | 0.341 | 0.066 | 0.033 |
| IEQ sumscore | Eye, in general | Parents | 60 | 0.342 | 0.067 | 0.033 |
| Moderate IEQ | Skin, in relation to school | Parents | 61 | 0.346 | 0.068 | 0.034 |
| Moderate IEQ | Respiratory, in general | Parents | 62 | 0.372 | 0.069 | 0.034 |
| Moderate IEQ | Lower respiratory, in general | Primary pupils | 63 | 0.450 | 0.070 | 0.035 |
| Moderate IEQ | Skin, in relation to school | Primary pupils | 64 | 0.472 | 0.071 | 0.036 |
| Poor IEQ | General, in relation to school | Secondary pupils | 65 | 0.482 | 0.072 | 0.036 |
| Poor IEQ | Eye, in general | Secondary pupils | 66 | 0.483 | 0.073 | 0.037 |
| Moderate IEQ | General, in relation to school | Primary pupils | 67 | 0.504 | 0.074 | 0.037 |
| Poor IEQ | Eye, in general | Primary pupils | 68 | 0.507 | 0.076 | 0.038 |
| Moderate IEQ | Eye, in general | Secondary pupils | 69 | 0.559 | 0.077 | 0.038 |
| Moderate IEQ | General, in general | Secondary pupils | 70 | 0.572 | 0.078 | 0.039 |
| IEQ sumscore | Skin, in relation to school | Parents | 71 | 0.579 | 0.079 | 0.039 |
| Moderate IEQ | Skin, in general | Secondary pupils | 72 | 0.604 | 0.080 | 0.040 |
| Moderate IEQ | Respiratory, in relation to school | Secondary pupils | 73 | 0.638 | 0.081 | 0.041 |
| Poor IEQ | Skin, in general | Secondary pupils | 74 | 0.670 | 0.082 | 0.041 |
| Moderate IEQ | Lower respiratory, in relation to school | Secondary pupils | 75 | 0.684 | 0.083 | 0.042 |
| Moderate IEQ | Lower respiratory, in relation to school | Parents | 76 | 0.699 | 0.084 | 0.042 |
| Poor IEQ | Lower respiratory, in relation to school | Secondary pupils | 77 | 0.719 | 0.086 | 0.043 |
| Moderate IEQ | Lower respiratory, in relation to school | Primary pupils | 78 | 0.720 | 0.087 | 0.043 |
| IEQ sumscore | Eye, in general | Primary pupils | 79 | 0.747 | 0.088 | 0.044 |
| IEQ sumscore | Eye, in general | Secondary pupils | 80 | 0.756 | 0.089 | 0.044 |
| Moderate IEQ | General, in general | Primary pupils | 81 | 0.758 | 0.090 | 0.045 |
| IEQ sumscore | Skin, in general | Secondary pupils | 82 | 0.759 | 0.091 | 0.046 |
| Moderate IEQ | Lower respiratory, in general | Parents | 83 | 0.776 | 0.092 | 0.046 |
| Poor IEQ | Eye, in relation to school | Secondary pupils | 84 | 0.823 | 0.093 | 0.047 |
| Moderate IEQ | Eye, in relation to school | Primary pupils | 85 | 0.842 | 0.094 | 0.047 |
| Moderate IEQ | Skin, in general | Primary pupils | 86 | 0.848 | 0.096 | 0.048 |
| Moderate IEQ | Lower respiratory, in general | Secondary pupils | 87 | 0.896 | 0.097 | 0.048 |
| Poor IEQ | General, in general | Secondary pupils | 88 | 0.955 | 0.098 | 0.049 |
| Moderate IEQ | Respiratory, in general | Secondary pupils | 89 | 0.973 | 0.099 | 0.049 |
| Moderate IEQ | Eye, in general | Primary pupils | 90 | 0.997 | 0.100 | 0.050 |

*Note*. Raw p-values lower than their corresponding critical values are statistically significant once adjusted for False Discovery Rate (FDR).

The formula to calculate critical values is the following: CV = (rank/number of tests)*FDR. For example, the first CV at FDR 0.10 was (1/90)*0.10=0.001. The dashed line between the 16^th^ and 17^th^ ranked p-values indicates the row below which the raw p-values are higher than the 0.05 critical values. The solid line between the 34^th^ and 35^th^ ranked p-values indicates the row below which the raw p-values are higher than the 0.10 critical values.

**Table S21.** The odds ratios and 95% confidence intervals for the associations between indoor environmental quality (IEQ) and symptoms of primary school pupils (n=8775, 99 school-buildings).

|  | **In generalᵃ** | | |  | **In relation to schoolᵇ** | | |
| --- | --- | --- | --- | --- | --- | --- | --- |
| **Symptom score** | **n (%)** | **OR** | **95% CI** |  | **n (%)** | **OR** | **95% CI** |
| *DV: Respiratory score* |  |  |  |  |  |  |  |
| IV: Latent classes***** of IEQ: |  |  |  |  |  |  |  |
| Good IEQ | 798 (20) | 1.00 |  |  | 240 (6) | 1.00 |  |
| Moderate IEQ | 708 (22) | **1.18** | **1.04 to 1.34** |  | 231 (7) | **1.25** | **1.02 to 1.53** |
| Poor IEQ | 355 (24) | **1.31** | **1.12 to 1.53** |  | 148 (10) | **1.73** | **1.37 to 2.19** |
|  |  |  |  |  |  |  |  |
| *DV: Lower respiratory score* |  |  |  |  |  |  |  |
| IV: Latent classes***** of IEQ: |  |  |  |  |  |  |  |
| Good IEQ | 99 (2) | 1.00 |  |  | 37 (0.9) | 1.00 |  |
| Moderate IEQ | 66 (2) | 0.88 | 0.64 to 1.22 |  | 25 (0.8) | 0.91 | 0.54 to 1.52 |
| Poor IEQ | 37 (3) | 1.39 | 0.97 to 1.98 |  | 20 (1.4) | 1.53 | 0.87 to 2.67 |
|  |  |  |  |  |  |  |  |
| *DV: Eye score* |  |  |  |  |  |  |  |
| IV: Latent classes***** of IEQ: |  |  |  |  |  |  |  |
| Good IEQ | 288 (7) | 1.00 |  |  | 165 (4) | 1.00 |  |
| Moderate IEQ | 226 (7) | 1.00 | 0.81 to 1.23 |  | 133 (4) | 1.03 | 0.79 to 1.34 |
| Poor IEQ | 113 (8) | 1.09 | 0.84 to 1.41 |  | 78 (5) | 1.32 | 0.96 to 1.81 |
|  |  |  |  |  |  |  |  |
| *DV: Skin score* |  |  |  |  |  |  |  |
| IV: Latent classes***** of IEQ: |  |  |  |  |  |  |  |
| Good IEQ | 332 (8) | 1.00 |  |  | 150 (4) | 1.00 |  |
| Moderate IEQ | 260 (8) | 0.98 | 0.82 to 1.18 |  | 111 (4) | 0.91 | 0.70 to 1.18 |
| Poor IEQ | 151 (10) | **1.30** | **1.05 to 1.61** |  | 78 (5) | **1.42** | **1.05 to 1.92** |
| *DV: General score* |  |  |  |  |  |  |  |
| IV: Latent classes***** of IEQ: |  |  |  |  |  |  |  |
| Good IEQ | 794 (19) | 1.00 |  |  | 453 (11) | 1.00 |  |
| Moderate IEQ | 632 (20) | 1.02 | 0.89 to 1.67 |  | 381 (12) | 1.06 | 0.89 to 1.27 |
| Poor IEQ | 374 (25) | **1.36** | **1.16 to 1.60** |  | 236 (16) | **1.44** | **1.16 to 1.79** |

*Note*. OR = Odds ratio, CI = Confidence intervals, DV = dependent variable, IV = independent variable. ᵃSymptoms reported without attribution to school environment (in general). ᵇSymptoms reported in relation to the school environment. * Number (percentage) of pupils experiencing symptoms is reported per each latent class of IEQ. The total number of pupils in each latent class is the following: Good IEQ: n = 4093 (47%), Moderate IEQ: n = 3201 (36%), Poor IEQ: n = 1481 (17%). All analyses were adjusted for pupils’ age, sex, asthma, hay fever, atopic rash, parental smoking, and attending Swedish-speaking school. Separate models were tested for each symptom score. Results highlighted in bold are statistically significant.

**Table S22.** The odds ratios and 95% confidence intervals for the associations between indoor environmental quality (IEQ) and symptoms of secondary school pupils (n=3410, 30 school-buildings).

|  | **In generalᵃ** | | |  | **In relation to schoolᵇ** | | |
| --- | --- | --- | --- | --- | --- | --- | --- |
| **Symptom score** | **n (%)** | **OR** | **95% CI** |  | **n (%)** | **OR** | **95% CI** |
| *DV: Respiratory score* |  |  |  |  |  |  |  |
| IV: Latent classes***** of IEQ: |  |  |  |  |  |  |  |
| Good IEQ | 237 (16) | 1.00 |  |  | 87 (6) | 1.00 |  |
| Moderate IEQ | 191 (16) | 0.99 | 0.73 to 1.36 |  | 81 (7) | 1.15 | 0.65 to 2.02 |
| Poor IEQ | 155 (20) | 1.23 | 0.89 to 1.69 |  | 77 (10) | 1.56 | 0.87 to 2.79 |
|  |  |  |  |  |  |  |  |
| *DV: Lower respiratory score* |  |  |  |  |  |  |  |
| IV: Latent classes***** of IEQ: |  |  |  |  |  |  |  |
| Good IEQ | 128 (9) | 1.00 |  |  | 27 (2) | 1.00 |  |
| Moderate IEQ | 108 (9) | 1.02 | 0.76 to 1.37 |  | 28 (2) | 1.20 | 0.49 to 2.94 |
| Poor IEQ | 84 (11) | 1.18 | 0.87 to 1.60 |  | 22 (3) | 1.19 | 0.46 to 3.11 |
|  |  |  |  |  |  |  |  |
| *DV: Eye score* |  |  |  |  |  |  |  |
| IV: Latent classes***** of IEQ: |  |  |  |  |  |  |  |
| Good IEQ | 246 (17) | 1.00 |  |  | 125 (9) | 1.00 |  |
| Moderate IEQ | 196 (16) | 0.93 | 0.75 to 1.17 |  | 84 (7) | 0.83 | 0.56 to 1.22 |
| Poor IEQ | 127 (17) | 0.92 | 0.72 to 1.17 |  | 71 (9) | 1.05 | 0.70 to 1.55 |
|  |  |  |  |  |  |  |  |
| *DV: Skin score* |  |  |  |  |  |  |  |
| IV: Latent classes***** of IEQ: |  |  |  |  |  |  |  |
| Good IEQ | 90 (6) | 1.00 |  |  | 16 (1) | 1.00 |  |
| Moderate IEQ | 75 (6) | 1.10 | 0.76 to 1.60 |  | 22 (2) | 2.06 | 0.99 to 4.26 |
| Poor IEQ | 42 (5) | 0.91 | 0.61 to 1.38 |  | 18 (2) | **2.40** | **1.19 to 4.83** |
|  |  |  |  |  |  |  |  |
| *DV: General score* |  |  |  |  |  |  |  |
| IV: Latent classes***** of IEQ: |  |  |  |  |  |  |  |
| Good IEQ | 844 (58) | 1.00 |  |  | 448 (31) | 1.00 |  |
| Moderate IEQ | 680 (57) | 1.07 | 0.85 to 1.35 |  | 313 (26) | 0.84 | 0.64 to 1.12 |
| Poor IEQ | 450 (59) | 0.99 | 0.77 to 1.28 |  | 251 (33) | 1.11 | 1.83 to 1.49 |

*Note*. OR = Odds ratio, CI = Confidence intervals, DV = dependent variable, IV = independent variable. ᵃSymptoms reported without attribution to the school environment (in general). ᵇSymptoms reported in relation to school environment. *Number (percentage) of pupils experiencing symptoms is reported per each latent class of IEQ. The total number of pupils in each latent class is the following: Good IEQ: n = 1447 (42%), Moderate IEQ: n = 1195 (35%), Poor IEQ: n = 768 (23%). All analyses were adjusted for pupils’ age, sex, asthma, hay fever, atopic rash, smoking, and attending Swedish-speaking school. Separate models were tested for each symptom score. Results highlighted in bold are statistically significant.

| Initial sample | |
| --- | --- |
| Primary pupils (3-6 grade):  Parents of primary pupils (1-6 grade):  Secondary pupils (7-9 grade): | 9835 pupils / 121 school buildings  3939 parents / 125 school buildings  3965 pupils / 38 school buildings |

| The schools with special education and school buildings with less than 10 responses per building were excluded | |
| --- | --- |
| Primary pupils (3-6 grade):  Parents of primary pupils (1-6 grade):  Secondary pupils (7-9 grade): | 9697 pupils / 108 school buildings  3769 parents / 94 school buildings  3804 pupils / 32 school buildings |

| The school buildings with missing expert evaluation of IEQ problems were excluded | |
| --- | --- |
| Primary pupils (3-6 grade):  Parents of primary pupils (1-6 grade):  Secondary pupils (7-9 grade): | 9138 pupils / 99 school buildings  3582 parents / 88 school buildings  3495 pupils / 30 school buildings |

| The participants with missing data on any of the covariates were excluded | |
| --- | --- |
| Primary pupils (3-6 grade):  Parents of primary pupils (1-6 grade):  Secondary pupils (7-9 grade): | **8775** pupils / **99** school buildings  **3540** parents / **88** school buildings  **3410** pupils / **30** school buildings |

**Figure S1. Flowchart describing how the school buildings and participants in three samples were selected for the study.**
